# Protein trans-splicing: optimization of intein-mediated GFP assembly as a model for the development of gene therapy

**DOI:** 10.3389/fbioe.2024.1488912

**Published:** 2024-11-20

**Authors:** Andrew Brovin, Ekaterina Minskaia, Matvei Sabantsev, Sergey Chuvpilo, Alexander Karabelsky

**Affiliations:** Gene Therapy Department, Research Center for Translational Medicine, Sirius University of Science and Technology, Sirius, Russia

**Keywords:** gene therapy, AAV, GFP, inteins, protein trans-splicing

## Abstract

Adeno-associated virus (AAV)-based gene therapy has become one of the key directions of modern translational medicine geared towards treatment of hereditary disorders by means of gene replacement. At the moment, about 5,000 different syndromes are associated with mutations in large genes, which presents a great problem due to the AAV packaging capacity of 5 kilobases. The main strategies for overcoming this obstacle were the creation of truncated gene versions, overloading the viral vector, and separate delivery of partial genetic material to restore the whole gene at the level of DNA, RNA, or protein. At present, genome editing via prime editors, most effectively delivered by AAV, relies on the intein pair used to restore the protein complex. The amazing integration speed of intein-based protein trans splicing technology makes it a versatile tool for a variety of applications, albeit not always successful on the first attempt. This study discusses the key points of working with Ssp, Npu, and Ava inteins of the DnaE group, known as the most effective for assembly of large proteins. Using green fluorescent protein (GFP) as a model, we demonstrate that the successful protein assembly requires not only cysteine at position C+1 but also certain aminoacid residues on either side in its immediate environment. Furthermore, the conformation of extein-intein composition, difficult to predict by computer modeling, has an additional effect, as demonstrated by experimental tests of the three split sites optimal in amino acid composition. The NpuDnaE variant demonstrated the highest kinetics of interaction between the N and C parts in the DnaE group of inteins. Optimization of conditions using NpuDnaE intein led to GFP assembly in 80% of transfected HEK293 cells and in 55% of AAV5-transduced cells, as demonstrated by flow cytometry. The efficiency of GFP assembly post-plasmid DNA transfection or AAV transduction of the HEK293 cell line was 15% higher than that of the ARPE19 cell line. We hope that the obtained data will facilitate the development of gene therapies for the treatment of hereditary disorders caused by mutations in large genes.

## 1 Introduction

**FIGURE 1:**
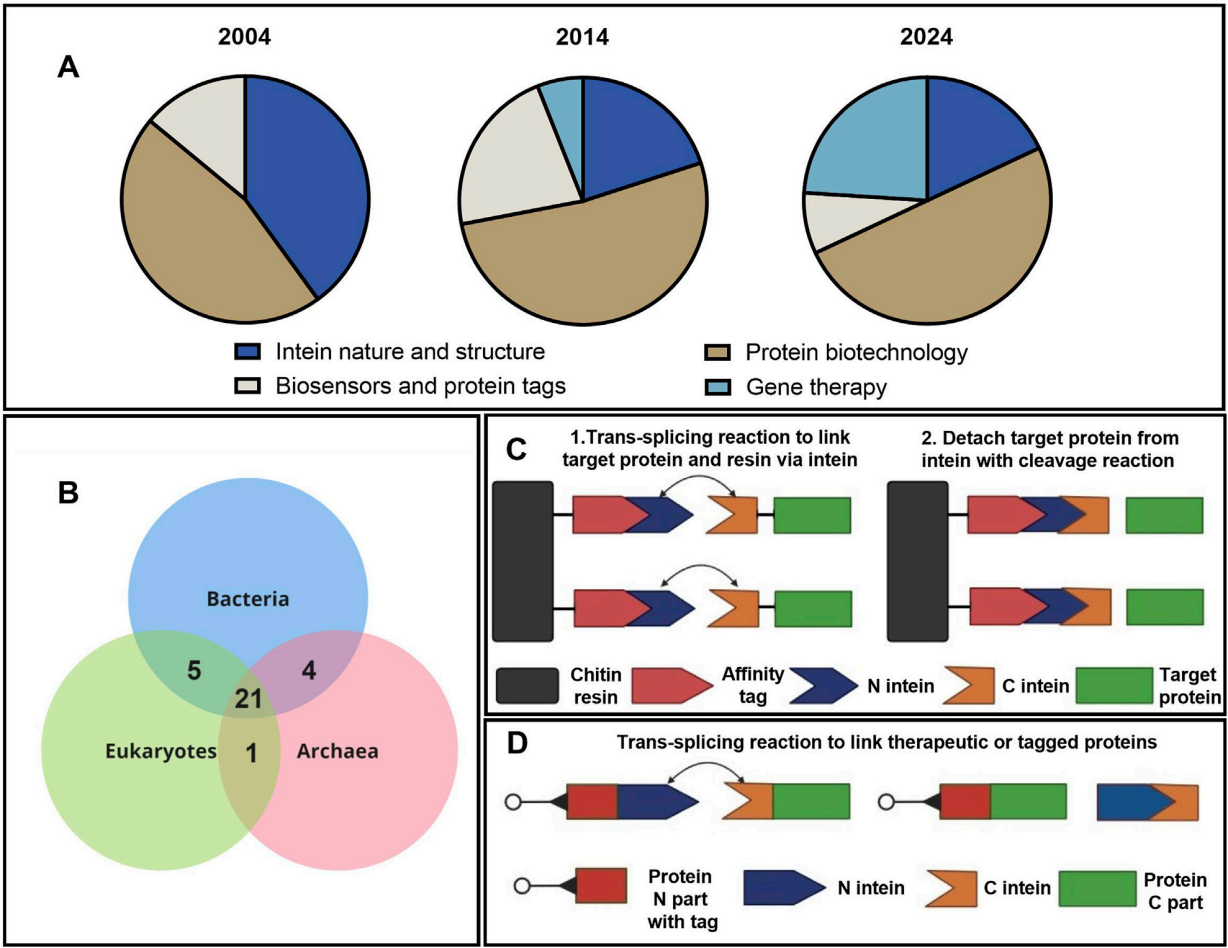
Applications of intein technology. **(A)** Distribution of studies using intein technology by topic and year (Supplementary Table S1). **(B)** Wien’s diagram depicts the number of host proteins of inteins common to the three domains of life (Pietrokovski, 2001). **(C)** Simplified schematic presentation of the IMPACT (NEB) chromatographic protein purification based on intein splicing. **(D)** Intein-based application of trans splicing technology for assembly and labeling of protein complexes.

Inteins have become powerful tools in synthetic biology, biotechnology, and biochemistry. These proteins were discovered by Bowman et al. (1988), and the mechanism of intein trans splicing was described by Perler et al. (1994). Over the last 30 years, the technology of protein trans splicing has been widely used for various applications, and about 70 studies with the keyword “intein” were published each year according to a PubMed search (Supplementary Table S1; PubMed, 2024). The chronological distribution of publications by topic has progressively changed, as can be seen in Figure 1A. With the discovery of trans splicing, common threads of research shifted from fundamental studies of structure, evolution, and biochemical features of inteins towards applied research in the area of protein purification and labeling, as well as gene therapy for hereditary diseases. These studies can be divided into several major theme blocks: the study of intein nature and evolution, their application for purification, isolation, and production of large proteins, the development of biosensors and protein detectors, and application in gene therapy for the treatment of hereditary diseases associated with mutations in large genes.

Evolutionary analysis of the inteins demonstrates that a major part of the intein-host ensemble is common to the three domains of life (Figure 1B). More interesting insights into intein evolution and structure can be found in the review by Pietrokovski (2001). One key insight from evolutionary studies points at specific composition of amino acids in intein sequences that form domains unique for these types of proteins. In fact, inteins consist of 10 motifs named A, N2, B, N4, C, D, E, H, F, and G. Each motif has a strictly fixed amino acid position that is crucial for splicing catalysis and a variable part that modulates the charge and hydropathy of the molecule. Thus, inteins can be identified by these catalysis centers in the functional Hint domain (A, N2, B, N4, F, and G) and traced through the common evolution of bacteria and unicell organisms. The evolutionary study of inteins is extensively discussed in a dozen review articles; the subject of this experimental article, however, relates to the application of inteins in biotechnology. A schematic presentation depicting the application of intein-splicing technology is shown in Figure 1C. Based on this approach, the IMPACT system was developed by NEB in 1997 (Chong et al., 1997). Another example of intein trans splicing technology used to link two separate proteins (Figure 1D) can overcome either the AAV packaging limitations or cytotoxicity of the full-length proteins (Gou et al., 2024). The addition of fluorescent or peptide tags allows for the visualization of protein assembly and transport (Cabantous et al., 2005; Jeon et al., 2018). Other useful and interesting examples of intein splicing applications can be found in the review (Nanda et al., 2020).

Inteins (derived from “internal protein”) are functional proteins of unicellular microorganisms that are capable of autocatalytic protein splicing. As a result, a whole protein is restored from its parts called exteins (“external protein”), followed by the excision of the auxiliary intein molecule.

**FIGURE 2:**
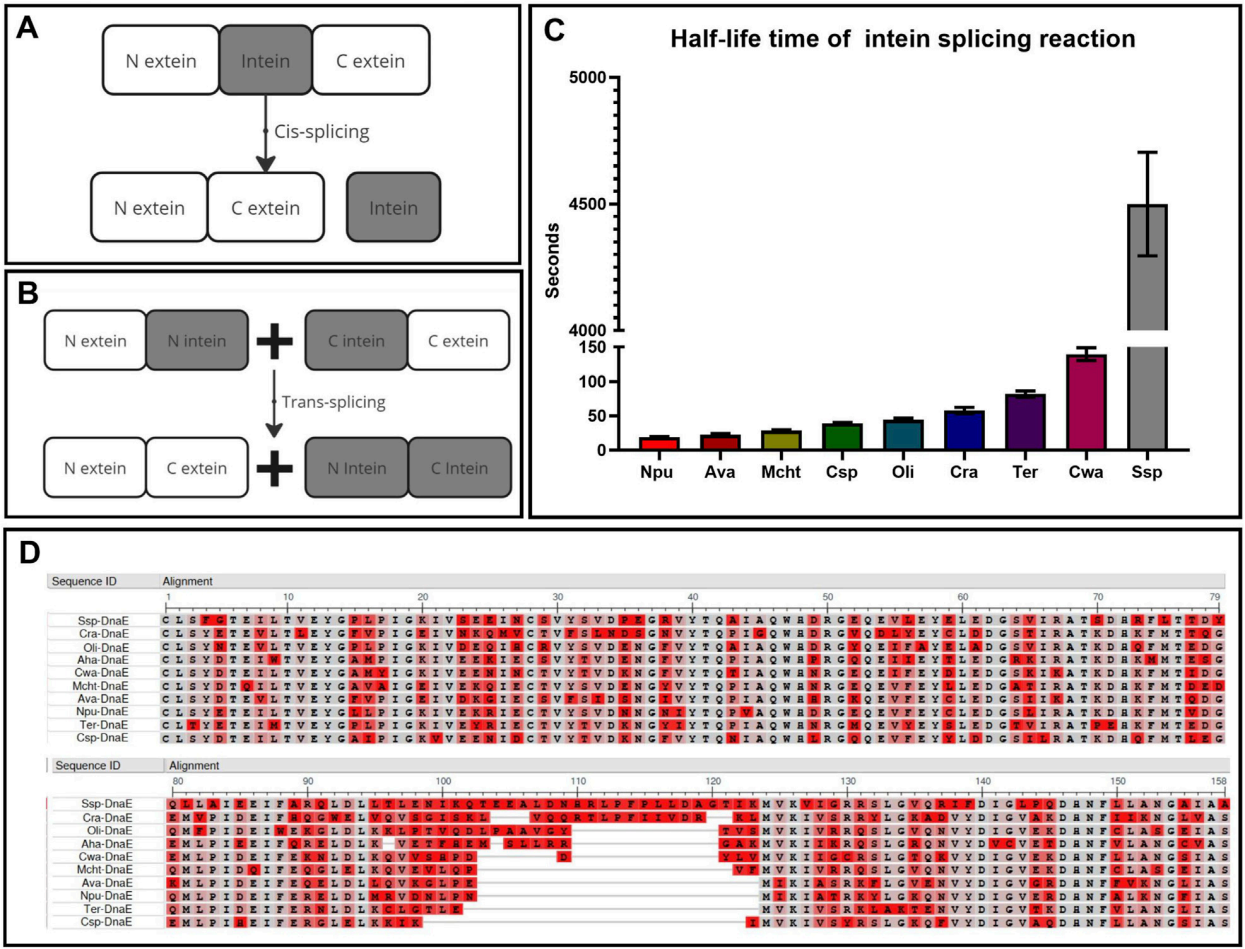
Key parameters for intein selection. **(A)** *Cis*-splicing to restore protein function and structure within one molecule. **(B)** *Trans*-splicing to restore protein function and structure within two separate molecules. **(C)** Comparison of intein kinetics within the DnaE enzyme group with the half-life time of interaction between intein parts (Shah et al., 2012). **(D)** Multiple alignment of intein sequences (N-part:1–123, C-part: 124–158) using COBALT NCBI software (COBALT NCBI, 2024), highlighting non-essential amino acids in red and conserved positions in gray.

There are two types of protein splicing depending on the exact location of splicing: *cis*- and *trans*-splicing. *Cis*-splicing, occurring within a single molecule via the excision of intein, which temporarily separates the functional domains of the protein (Figure 2A), is most commonly used as a self-excision process to increase the yield of the purified protein (Warren et al., 2013; Jiang et al., 2015). *Trans*-splicing, on the other hand, occurs between two separate molecules (Figure 2B) and is commonly used for the separate delivery and assembly of large protein complexes (Esposito et al., 2022) or as a biosensor (Cabantous et al., 2005; Jeon et al., 2018).

This technology offers several significant advantages over other forms of separate delivery and assembly of large DNA and RNA fragments. Firstly, the highly specific enzymatic interaction of inteins by the “key-lock” mechanism occurs much faster and more specifically than the donor-acceptor mechanisms of DNA and RNA trans-splicing without any off-target effects that may happen more frequently after gene recombination. Secondly, the process is autocatalytic, caused only by the composition of N extein and intein amino acids that trigger the reaction by the chemical nature of the trans-splicing reaction, meaning no additional substrates or catalysts are required for the reaction. The only factors accelerating this reaction are pH and temperature, which do not affect the mechanism itself but influence the speed of molecular movement. Thirdly, if the efficiency of the reaction is influenced only by the amino acid composition, it is important to understand the degree of contribution of these amino acids. For example, the amino acid sequence alignment of inteins from one group of the third polymerase E subunit in different organisms (Figure 2D) demonstrates their high degree of conservation. Sixty percent of the N- and C-termini are completely identical at the sites of active and catalytic centers (highlighted in gray), while the variable amino acids (highlighted in red) surround the active center and slightly increase the charge of the molecule, providing greater affinity and speed of interaction between the N-positively and C-negatively charged parts (Figure 2C) (Shah et al., 2012).

Special attention should be given to the first three N-terminal and the last three C-terminal amino acids, which possess the key catalytic properties. The significance of these intein residues was highlighted in several studies (Nichols and Evans, 2004; Ludwig et al., 2008; Wasmuth et al., 2013; Stevens et al., 2016), pointing at the crucial influence of the composition of extein residues on the N terminus, which initializes splicing reactions with an N-X shift caused by energy conversion from the unstable conformation of N extein and N intein to the stable form of a highly reactive etherized N molecule that attacks the C part. The C terminus containing free and reactive OH or SH groups placed on cysteine, serine, threonine, and methionine is required for further processing of nucleophilic attack. The completion of the trans etherical reaction requires asparagine, aspartic acid, or glutamine at the end of C-intein to ligate two protein fragments. Thus, cysteine, serine, threonine, and methionine at the C+1 extein and the last asparagine, aspartic acid, or glutamine aminoacid at the C part of the intein are crucial for processing of the slicing reaction (Starokadomskiĭ, 2007; Cheriyan et al., 2013). In addition, cis conformation of N extein and intein is required for triggering all splicing reactions (Klabunde T et al., 1998). Thus, the N part of extein is a more variable and flexible part of the trans etherical reaction, while the C part is much more fixed and constant with only three variables at all.

The effect of the secondary structure of the target protein at the split site on the successful assembly can usually be determined experimentally. While secondary structure and interaction of intein parts can be predicted by several machine learning tools (Schmitz et al., 2024), it is important to evaluate the efficiency of the predicted intein trans-splicing experimentally (Cheriyan et al., 2013).

To summarize, intein-mediated splicing technology is crucial for gene therapy applications seeking to restore the function of large proteins. Detailed discussion about the current state and limitations of AAV-based gene therapy, as well as strategies used to overcome the limitations, can be found in the review by Kolesnik and co-authors (2024). The focus of our experimental study was on the development of an intein trans-splicing methodology using GFP protein as a model and several well-studied and characterized inteins as examples with the aim of optimizing the *trans*-splicing process. The following criteria were considered when choosing candidate inteins: splicing type, the size of polypeptides, and the rate of interaction between them. While this technology has a variety of applications, our current and future focus is on its potential use for protein replacement therapy for hereditary diseases.

## 2 Materials and methods

### 2.1 Design, assembly and cloning of genetic constructs

The amino acid sequences of the SspDnaE, NpuDnaE, and AvaDnaE inteins were obtained from the InBase database (Perler, 2002). Sequence coding for GFP was PCR-amplified using primers incorporating BamHI and HindIII restriction sites (Supplementary Table S2) and plasmid pGFP-N3 (Clontech, Takara Bio Inc., Japan) as a template. Two optimal cleavage sites (47 and 69) and a negative control (127) were selected using the previously described algorithm (Cheriyan et al., 2013) and data from the spatial model 6YLQ (RCSB PDB, 2024) based on the highest and lowest score. N and C fragments of the intein were added in frame to GFP parts. Codon optimization of the amino acid sequence was performed using the back-translation function of the Benchlink software (Benchlink, 2024) to reduce the GC content of the nucleotide sequence.

Two-step overlapping PCR (Young L. and Dong Q., 2004) was used to assemble constructs with the Tersus Plus PCR kit (PK221, Evrogen, Russia). A typical PCR reaction contained 4 µL of 5 × Tersus Red buffer, 0.5 µL of 10 mM dNTP mix, 0.5 µL of 50 × Tersus polymerase mix, 2 µL of 10 pM oligonucleotide mix, 12 µL of dH2O, and 1 µL of DNA template. The following amplification program was used for PCR reaction on the T100 Thermal Cycler (1861096, BioRad, United States): initial denaturation at 95°C for 5 min, followed by 30 cycles of denaturation at 95°C for 10 s, annealing at 60°C for 20 s, and elongation at 72°C for 30–60 s, depending on the size of the PCR product. The fragment assembly was performed with oligonucleotides, followed by amplification of the final PCR product. A similar overlapping method was performed with the two GFP fragments. The PCR products were purified using a CleanUp Mini kit (BC023L, Evrogen, Russia) according to the manufacturer’s instructions and cloned using the above-mentioned restriction sites. All constructed plasmids were verified by restriction digestion and sequencing (ABI 3500 sequencer, Thermo Fisher Scientific Inc., United States).

pAAV-MCS Expression Vector of the AAV5 CMV Expression System kit (VPK-410-SER5, Cell Biolabs, United States) was used for construction of the pAAV-GFP vector to produce AAV5-GFP. The PCR product, amplified with primers incorporating BamHI and HindIII restriction sites and 6 × His tag sequence on each 3′end, was cloned into pAAV-MCS vector digested with these restriction enzymes. Positive clones were confirmed by restriction digestion and sequencing.

### 2.2 Production and purification of AAV5-GFP

To produce AAV5-GFP, suspension HEK293 cells were transfected with pAAV-GFP, pAAV-RC5, and pHelper plasmids according to the previously described protocol (Tumaev et al., 2024). Briefly, the plasmids, used at 1.5 µg per 1 × 10^6^ cells at a molar ratio of 2:2:5 (pAAV-MCS:pHelper:RepCap 2/5), were mixed with PEI-MAX (24765, Polysciences Inc., United States) at a ratio of 1:5 (DNA:PEI). After 24 h, EmCD HEK293 Plus Feed (P30310.0001, Eminence, China) was added to the suspension, and the cells were further incubated for 96 h at 180 rpm, 37°C, and 80% humidity.

Viral particles were isolated by the addition of 10% Tween 20 (P1379, Sigma-Aldrich-Merck Life Science LLC, United States) to the cell suspension to a final concentration of 0.05%, followed by incubation for 1 h. Then, 1M MgCl_2_ (M9272, Sigma-Aldrich-Merck Life Science LLC, United States) was added to the final concentration of 1 mM and benzonase CAS 9025-65-4 (3549.0250, Dia-M, Russia) to a final concentration of 30 units/mL, and incubation continued for 1 h. Following centrifugation at 3500 g for 10 min, 1 g of Celite^®^ 503 diatomaceous earth (22151, Sigma-Aldrich-Merck Life Science LLC, United States) per 100 mL of supernatant was added, and supernatant was filtered through a 0.22 µm sterile filtration system (344001, Nest, China) and concentrated on a Vivaflow 200 TFF cassette PES tangential flow filtration system with a 100 kDa filter (VF20P4, Sartorius AG, Germany). For affinity chromatography, the prepared viral particle solution was filtered through a PES syringe filter with a 0.22 µm pore size and then applied to a HiTrap AVB Sepharose HP column (28411211, Cytiva, Sweden) with an AVB Sepharose High Performance sorbent (28411210, Cytiva, United States) and an NGC Discover 10 medium pressure chromatography system (7880009, BioRad, United States). Chromatography was performed according to the manufacturer’s instructions using equilibration (20 mM Tris, 200 mM NaCl, pH 7.8), wash (20 mM Tris, pH 8.0), elution (50 mM Glycine, pH 2.7), regeneration (0.1 M citrate, pH 2.1) and storage (10 mM NaOH, pH 2.1) buffers.

To evaluate the efficiency of chromatography and determine the final concentration of viral particles, real-time PCR was performed according to the previously published protocol (Aurnhammer et al., 2012). Samples were treated with DNase I and proteinase K as follows: 2 µL of 10 × DNase buffer (100 mM Tris-HCl (pH 7), 30 mM MgCl_2_, 30 mM CaCl_2_), 1 µL of DNase I (EM-1250, Biolabmix, Russia), 2 µL of AAV-GFP, and 15 µL of H_2_O were mixed in a total volume of 20 µL. The mix was incubated for 30 min at 37°C, and the enzyme was inactivated for 10 min at 55°C. Then, 20 µL of proteinase K (EP-1200, Biolabmix, Russia) at a concentration of 0.1 units/μL were added to the mixture. The mix was incubated for 55 min at 55°C and inactivated for 5 min at 95°C.

Quantitative PCR (qPCR) was performed with forward (5′-GGA​ACC​CCT​AGT​GAT​GGA​GTT-3′) and reverse (5′-CGG​CCT​CAG​TGA​GCG​A-3′) primers and probe (5′-FAM-CACTCCCTCTCTGCGCGCTCG-BHQ1-3′) (Aurnhammer et al., 2012).

Linearized plasmid pAAV-MCS was used at a concentration of 10 ng/μL (the highest concentration of 1 × 10^9^ gene copies/mL) with serial 10-fold dilutions down to 1 pg/μL (the lowest concentration of 1 × 10^4^ gene copies/mL) for the construction of the standard curve. The PCR master mix (enough for 110 reactions) contained the following components: 800 µL of BioMaster HS-qPCR Hi-ROX ready mix (MHR020-2040, Biolabmix, Russia), 110 µL of H_2_O, 5 µL of each forward and reverse primer, and probe at a concentration of 100 pmol/μL. The analysis was performed using the StepOnePlus real-time PCR system (437659, Thermo Scientific, United States) with the Quantification-Standard Curve measurement mode in the StepOneSoftWare V2.3 software. Acceptance criteria for the analysis were a linearity of the standard curve greater than 0.9, reaction efficiency in the range of 85%–100%, a standard deviation of the threshold cycle for a single sample of no more than 0.2, and sample concentration values belonging to the linear range of determined concentrations. The obtained data were used for the transduction of cells with prepared viral particle preparations at the selected dose.

### 2.3 Cell lines

The HEK293 (human embryonic kidney) cell line was obtained from the European Collection of Authenticated Cell Cultures (85120602, ECACC, 2024). ARPE-19 (human retinal pigment epithelium) cell line was gifted by the Cell Culture Collection of Koltzov Institute of Developmental Biology of the Russian Academy of Sciences (ARPE-19, IDB RAS, 2024). The FbP13 (human primary fibroblasts) cells were gifted by the All-Russian collection of biological samples of hereditary diseases of the Research Centre of Medical Genetics (200247, RCMG, 2024). Each cell line was maintained in DMEM/F12 media (С470, PanEco, Russia) supplemented with 10% FBS (S181H-500, Biowest, France). All adherent cell lines were maintained in a humidified incubator at 37°C and 5% CO2 and grown to 80% confluence. No additional supplements or antibiotics were added to the media.

### 2.4 Transfection and transduction of adherent cells

5 × 10^5^ cells were transfected with 2 µg of total plasmid DNA and PEI reagent at a DNA:PEI ratio of 1:5. Twenty-four hours post-transfection, the media was changed to 2 mL of fresh DMEM/F12 (5% FBS) and the cells were incubated for 48 h.

Transduction of 3 × 10^4^ cells per well of a 96-well plate with AAV5, delivering two parts of GFP protein, was carried out at 1 × 10^5^ Vg/cell each in a final volume of 100 µL. Twenty-four hours post-transduction, the media was changed to 200 µL of fresh DMEM/F12 (5% FBS) and the cells were incubated for an additional 48 h.

### 2.5 Live cell microscopy

An IncuCyte S3 live cell microscope was used for qualitative and semi-quantitative fluorescence analysis (Sartorius AG, Germany), with automatic scans taken every 2 h in two optical channels (bright field and green) for 48 h. The number of green objects per image was used as the primary metric for analysis, and background fluorescence was subtracted. This analysis determined the dynamics of fluorescence accumulation.

### 2.6 Flow cytometry

The efficiency of transfection and transduction was determined by the percentage of GFP-positive (GFP+) cells and median fluorescence intensity (MFI). To prepare the samples for analysis, the cells were trypsinized, washed twice with 500 μL PBS, and resuspended in 250 µL chilled FACS buffer (1 × PBS, 5% FBS, 1 mM EDTA). The data was recorded on the CytoFlex B2-R2-V0 flow cytometer (A00-1-1102, Beckman Coulter, United States) using CytExpert software v1.2 gating on single FITC-positive live cells. Further analysis was carried out using gating strategy (Supplementary Figures S5, S6).

### 2.7 Western blotting

Seventy-two hours post-transfection or transduction, the cells were resuspended in 500 µL PBS and 1 × complete protease inhibitor cocktail (11697498001, Roche, Switzerland). The cell suspension was homogenized using a sonicator for 40 s and centrifuged for 10 min at 13,000 g. The samples were prepared by mixing 45 µL of cell lysates with 15 µL of 4 × Laemmli buffer (4% SDS, 10% 2-mercaptoethanol, 20% glycerol, 0.004% bromophenol blue, and 0.125 M Tris-HCl pH 6.8) in a total volume of 60 µL. After incubation at 100°C for 10 min, 25 µL of each sample as well as 5 µL of Precision Plus Protein™ Kaleidoscope™ Prestained Protein Standards (1610375, BioRad, United States) were loaded onto 12.5% PAGE gel and run at 150 V for 1 h in a 1 × running buffer (25 mM Tris base, 190 mM glycine, 0.1% SDS, pH 8.3). The proteins were transferred onto pre-activated PVDF membrane (Immobilon®-P, IPVH00010, Merck Life Science LLC, United States) using the Trans-Blot Turbo Transfer System (1704150, BioRad, United States) and buffer (25 mM TrisOH, 190 mM glycine, 0.1% SDS, pH 8.3). The membrane was blocked in TBS-T buffer (Tris-buffered saline, 0.1% Tween 20) containing 5% dry milk (1706404, BioRad, United States) for 2 h, incubated with either primary anti-GFP (01101231265, Evrogen, Russia) or anti-6 × His (MCA5995P, BioRad, United States) antibodies overnight (1:2000 dilution in 5% dry milk), washed in TBS-T three times, and incubated with HRP-conjugated secondary antibody (HAF008, R&D Systems, United States) for 2 h. The washed membrane was subjected to enhanced chemiluminescence (ECL, 1705062, BioRad, United States), and the proteins were visualized using the ChemiDoc™ MP Imaging System (12003154, BioRad, United States). After stripping the membrane, the procedure was repeated with antibodies against GAPDH (8G4R, Hytest, Russia) used for normalization as reference protein. Densitometry analysis of images was performed using ImageJ 1.54 g software with built-in gel analysis tool. Normalization was performed as the ratio of the target protein to housekeeping protein densitometry values.

### 2.8 Statistical analysis

Statistical analysis of the normal distribution of samples and the confidence interval of differences for rejection of the null hypothesis was performed using GraphPad Prism 9.3.1 software (ordinary one-way ANOVA, Sidak multiple comparison test). Results are presented as mean ± standard deviation of 2-3 biological replicates, confidence interval: level of difference is significant (*) p-value <0.05, (**) p-value <0.01, (***) p-value <0.001, (****) p-value <0.0001, not significant (ns) p-value >0.05.

## 3 Results

### 3.1 Project pipeline

**FIGURE 3:**
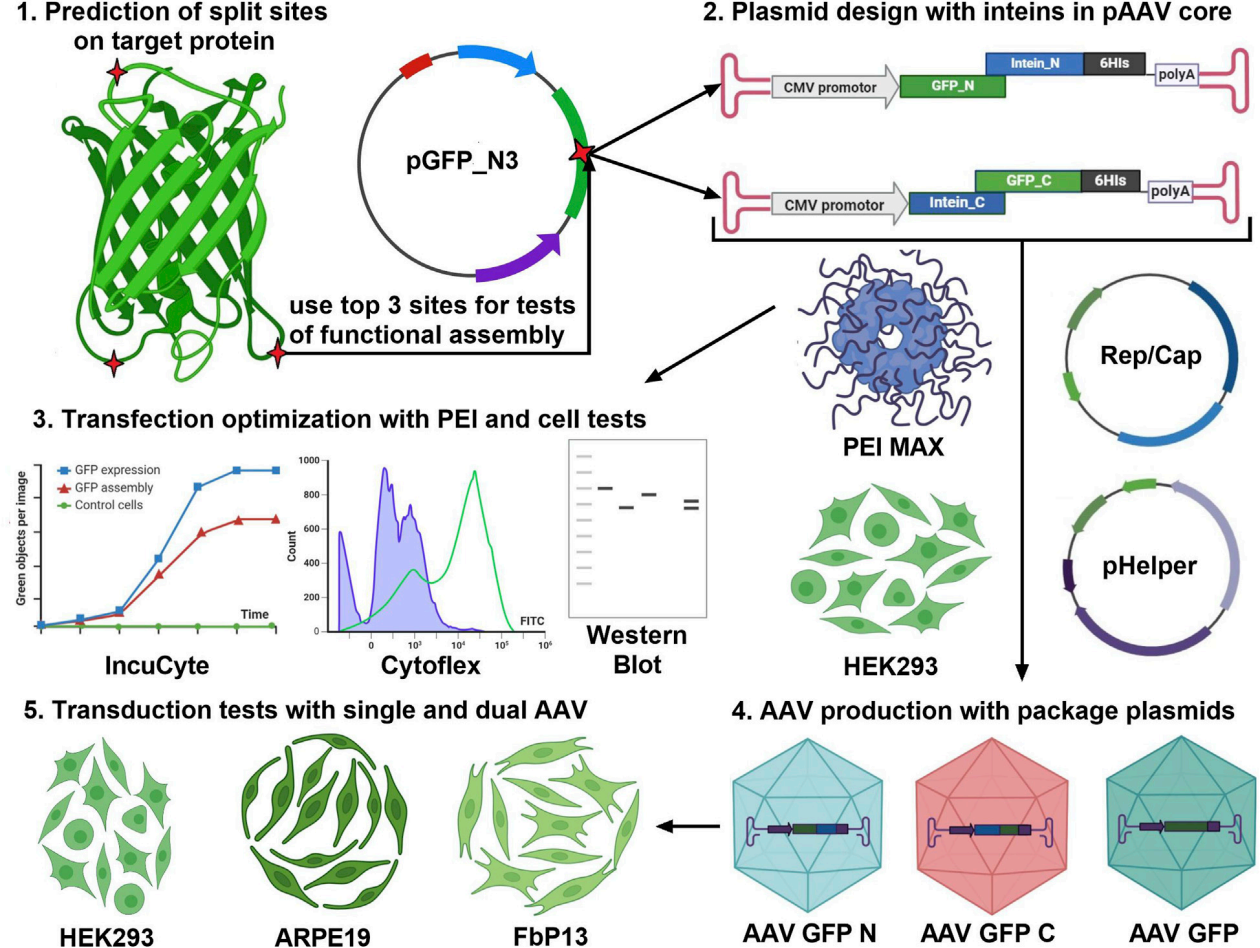
Project pipeline for the development of gene therapy products based on the intein trans splicing technology. The first step is to analyze the target protein molecule (GFP is shown as an example) using a splicing site search algorithm (Cheriyan et al., 2013) and paying attention to protein secondary structure. The top three predicted sites should be used for the design of transient expression plasmids and pAAV transfer plasmids for AAV transduction. Efficiency of GFP assembly post-AAV transduction is assessed in three human cell lines of different origin.

The first important step to consider when assembling proteins is to choose the type of protein splicing and intended application, as shown in Figure 3. *Cis*-splicing inteins SspDnaB, usually used for selective protein purification (Jiang et al., 2015), and trans-splicing inteins Rma, DnaB, and Cfa of the DnaE group can be used for protein complex assembly and biosensor activation (Li et al., 2023). The second step involves the selection of a specific version of intein based on the length of the amino acid sequence and the kinetic parameters of the N and C parts of the interaction. It should be noted that the kinetics of splicing play a key role in the efficiency of the target protein restoration. It is also advisable to do the secondary structure prediction using computer modeling. However, *in silico* modeling of trans-splicing requires high-load computational dynamic analysis, and the effect of secondary structure on trans splicing should be tested experimentally. The most useful predictive information can be obtained by split site analysis, which relies on an experimental data table of the impact of each of the six amino acids surrounding the split site. The fourth point to consider is that the sequence encoding N part of the protein of interest, N intein, and a specific tag (such as 6 × His or Flag) should comprise one open reading frame. The final construct should be reverse-translated using codon optimization parameters. The last experimental step estimates the impact of secondary structure, trans splicing assembly, and protein activity. Functional tests demonstrate that protein activity was not lost as a result of the split-site selection procedure and incorrect confirmation. In the case of fluorescent proteins, their function is easily confirmed by the presence of fluorescence, but confirmation of the activity of therapeutic proteins requires specific tests. Finally, dose-dependent AAV transduction efficiency as well as separate gene fragment delivery into specific cell types and the assembly of functional proteins need to be evaluated by flow cytometry, Western blotting, and functional tests. The human embryonic kidney cell line HEK293 is recommended for the initial optimization of transfection and transduction conditions, followed by other relevant cell lines that may be difficult to transfect or transduce. This pipeline is universal for the development of AAV-based therapies relying on intein trans-splicing for the assembly of large genes.

### 3.2 GFP amino acid sequence analysis

Inteins of the DnaE group were chosen as the most studied and shortest natural trans-splicing inteins (Ellilä et al., 2011; Shah et al., 2012). Each intein group has a unique requirement for each of the six amino acid residues at a split site due to their effect on the overall intein catalytic activity (Ellilä et al., 2011). The presence of cysteine at position +1 is critical for the DnaE group of inteins. More detailed alanine screening (Ludwig et al., 2008) of short peptides showed the impact of cysteine presence as well as the additional influence of asparagine (N) at position −1. Another study (Cheriyan et al., 2013) of NpuDnaE inteins with 485 variations of every amino acid at each N-3 and C+3 position gave a numeric estimation of each amino acid’s impact on the trans-splicing process. In this study, an algorithm for protein partition and sequence analysis was based on this experimental data table. Unfortunately, the available data does not allow for the prediction of the impact that secondary structure may have on the restoration of functional conformation.

While more advanced computational analysis has been done recently (Schmitz et al., 2024), earlier studies tested the efficiency of splicing site-based assembly experimentally using plasmid co-transfection (Li et al., 2023). Evaluation of GFP split sites done with the Python 3.0 subfunction written in Jupiter notebook using the existing table (Cheriyan et al., 2013) revealed two optimal sites at positions 69/70 and 47/48 and one negative control site at position 127/128 (Table 1).

**TABLE 1:** Sequence analysis of GFP protein partitioned by 6-amino acid sites. Each site was evaluated with an algorithm based on previously published data (Cheriyan et al., 2013) and ranked by score, which indicates the impact of amino acid composition on trans-splicing efficiency of DnaE inteins. Sites highlighted in green have the highest, in yellow - lower, and in gray - lowest predicted efficiency of trans-splicing. Sites highlighted in red were used for plasmid design with SspDnaE intein.

Rating position	Sequence	Site score	Site position	Secondary structure	References
1	GVQCFS	55.64	69/70	ALFA-HELIX	Tornabene et al. (2019)
2	KFICTT	52.32	47/48	BETA-SHEET	
3	LPVPWP	34.32	55/56	ALFA-HELIX	
4	KSAMPE	30.25	86/87	ALFA-HELIX	Khoo et al. (2020)
5	TLGMDE	26.87	228/229	NON STRUCTED	
6	RDHMVL	26.37	217/218	BETA-SHEET	
7	PDHMKQ	24.85	77/78	ALFA-HELIX	
8	VYIMAD	22.42	152/153	BETA-SHEET	
9	GEGEGD	22.29	33/34	BETA-SHEET	
10	GDVNGH	19.7	21/22	NON STRUCTED	
11	ADKQKN	19.64	156/157	NON STRUCTED	Kanno et al., 2011 Tasfaout et al. (2024)
76 positions are skipped					
87	KGIDFK	5.93	127/128	NON STRUCTED	Ozawa (2006)
98 positions are skipped					
185	TTLTYG	2.8	64/65	NON STRUCTED	Khoo et al. (2020)
49 positions are skipped					
234	LVELDG	0.82	17/18	BETA-SHEET	

**FIGURE 4:**
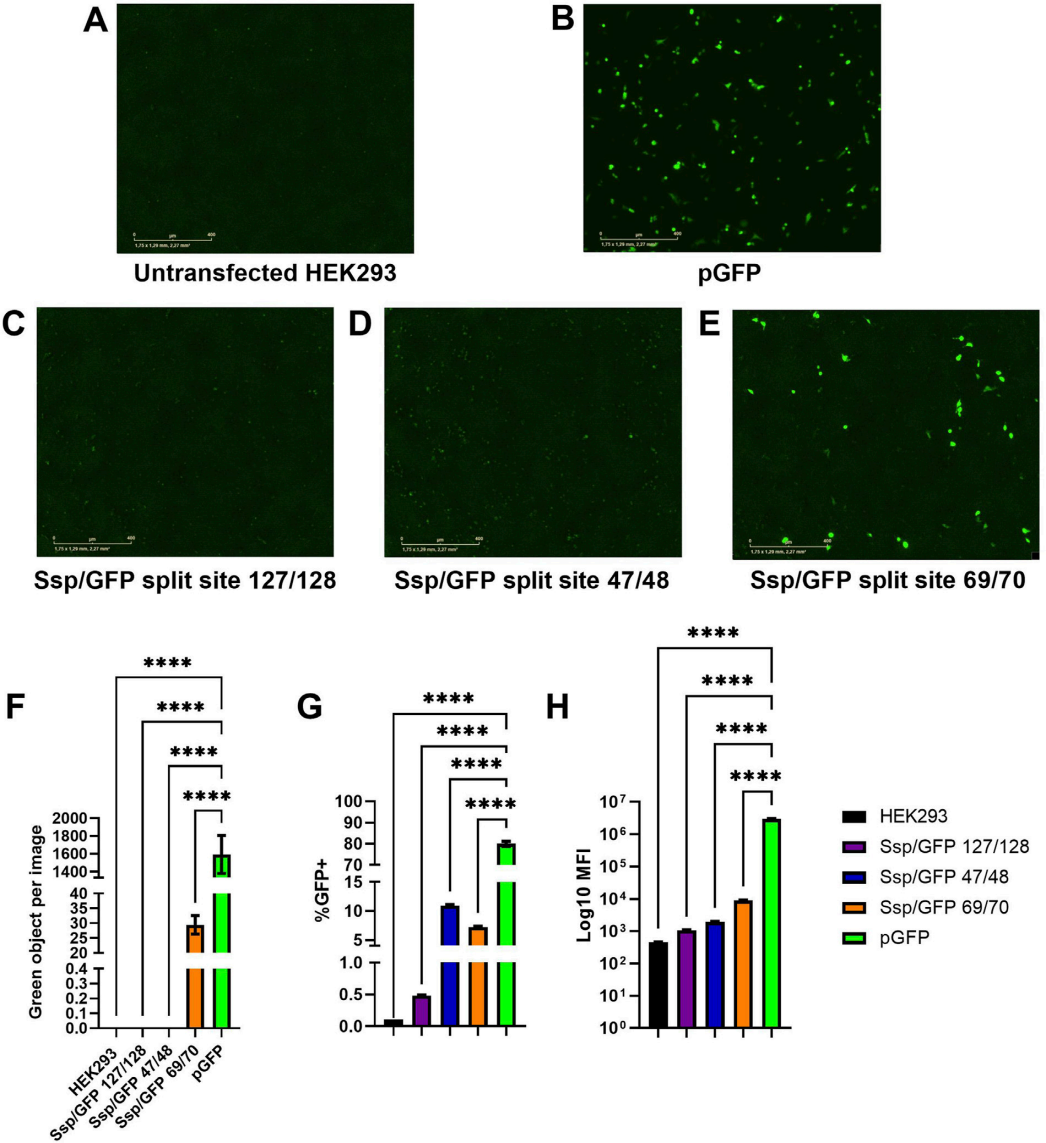
Assessment of predicted GFP split sites at positions 47/48, 69/70, and 127/128. **(A–E)** Images of green fluorescence in HEK293 cells at 48 h post-transfection with 2 µg of total DNA (2:1 PEI:DNA ratio) assessed by live cell microscopy with IncuCyte. The cells were transfected with plasmids expressing full-length GFP **(B)**, GFP split at 127/128 position and SspDnaE intein **(C)**, GFP split at 47/48 position and SspDnaE intein **(E)**, and GFP split at 69/70 position and SspDnaE intein **(D)**. **(F)**. The level of fluorescence intensity (green objects per image). The percentage of GFP+ cells **(G)** and the median fluorescence intensity [MFI, **(H)**] were evaluated by flow cytometry at 72 h post-transfection. (****) p-value <0.0001.

GFP assembly as a result of co-transfection of Ssp/GFP plasmids encoding the parts of GFP reporter protein and SspDnaE intein was assessed by fluorescent microscopy (Figures 4A–E) and flow cytometry (Figures 4F–H) at 48 h post-transfection.

Untransfected HEK293 cells were used as a negative control to assess autofluorescence (Figure 4A) and to set negative gates in flow cytometry. Cells transfected with a pGFP plasmid encoding the full-length GFP were used as a positive control of transfection and GFP expression for comparison with intein-assembled versions divided at three split sites: 127/128 (C), 47/48, (D), and 69/70 (E). Green fluorescence intensity of the split site 69/70 variant was considerably higher than that of the other two variants, as demonstrated by IncuCyte data (Figure 4F). The results of flow cytometry analysis carried out at 72 h post-transfection demonstrated that 7.2% of cells transfected with the 69/70 split site variant and 10.9% of cells transfected with the 47/48 split site variant were GFP+, as compared to the positive control (80% of cells). Importantly, these differences were statistically significant (p-value <0.0001). MFI values gradually increased from 1.08 × 10^3^ (127/128 split variant) to 1.98 × 10^3^ (47/48 split variant) to 9.06 × 10^3^ (69/70) split variant), additionally supporting the data obtained in IncuCyte and confirming the 69/70 split site variant as the most efficient one for protein assembly. Statistically significant differences were shown between all split variants and pGFP-transfected cells (Figure 4G). The 127/128 split site used as a negative control for the splicing reaction was indistinguishable from untransfected cells by either microscopy or flow cytometry, demonstrating that an incorrect combination of amino acids can completely abolish the trans-splicing reaction even in the presence of an otherwise active intein and unstructured protein surface.

### 3.3 Optimization of transfection for intein trans-splicing in HEK293 cells

Only 7.2% of transfected cells expressed GFP after intein-mediated assembly, according to the flow cytometry analysis, demonstrating the dramatically low efficiency of the trans-splicing reaction. Successful biomedical applications, however, require protein assembly in at least 50% of the cell population. With the aim of achieving this goal, the following parameters were further optimized using the two-plasmid system of SspDnaE intein and 69/70 split GFP: total amount of plasmid DNA, molar ratio between PEI and DNA, and the order in which the key components were added.

The amount of transfected plasmid DNA varied because the individual impact of each part on the protein trans-splicing reaction was unknown. Individual impact of the N- and C-terminal parts on trans splicing reaction was evaluated by Western blotting. The cells were transfected with 0.25, 0.5, 1, 2, or 3 µg of total DNA at a 2:1 PEI:DNA molar ratio (Supplementary Figure S1). As expected, while no fluorescence could be observed in untransfected cells and cells transfected with lower amounts of DNA (Supplementary Figures S1A–E), its intensity increased significantly when 2 and 3 µg of total DNA were used (Supplementary Figures S1E, F), as demonstrated by both IncuCyte microscopy (Supplementary Figure S1G) and flow cytometry (Supplementary Figures S1H–I). The IncuCyte analysis of green objects with background compensation revealed that the level of fluorescence in cells transfected with 2 µg (86 objects per image) and 3 µg (35 objects per image) of DNA was significantly higher than that of cells transfected with 0.25 and 0.5 µg of plasmid DNA (almost zero) (Supplementary Figure S1G). In confirmation of live microscopy data, 6.8% and 4.9% of cells transfected with 2 and 3 µg of total DNA, respectively, were GFP+, as shown by flow cytometry analysis (Supplementary Figure S1H). Additionally, the most significant differences in MFI values were observed in cells transfected with 2 µg (4730 units) and 3 µg (3300 units) of total DNA, which were 2.5 and 1.5 times higher than the negative control (2300 units). Therefore, 2 µg of plasmid DNA, the optimal amount that led to higher efficiency of GFP assembly and fluorescence in a two-plasmid system, was used for further optimization of the PEI:DNA ratio.

In order to further improve transfection efficiency and intein-mediated GFP assembly, an optimal molar ratio of transfection components was tested experimentally. The transfection mix contained 2 µg of plasmid DNA and PEI at the following PEI:DNA molar ratios: 1:1, 2:1, 3:1, 5:1, and 8:1. As can be seen in Supplementary Figure S2, both the number of GFP+ cells and fluorescence intensity gradually increased proportionately to PEI amounts (Supplementary Figures S2A–E), with the highest amount of PEI, not unexpectedly, resulting in cytotoxicity (Supplementary Figure S2F). As shown by IncuCyte analysis, the levels of fluorescence intensity were similar in all samples (43–51 green objects per image) with the exception of 2:1 PEI:DNA transfection (92 green objects per image) (Supplementary Figure S2G). It is worth noting that the microscopy analysis is more sensitive to false signal detection due to cell aggregation; therefore, flow cytometry analysis was used to further confirm the results. The number of GFP+ cells gradually increased from 10.6% (PEI:DNA ratio of 1:1) to 44% (PEI:DNA ratio of 3:1) with increasing amounts of PEI used for transfection. The number of GFP+ cells did not change significantly when higher amounts of PEI were used (46% for a 5:1 ratio and 51% for an 8:1 ratio) (Supplementary Figure S2H). The mean fluorescent intensity values increased from 1794 (1:1 ratio) to 3036 (2:1 ratio), 5014 (3:1 ratio), and 6656 (5:1) (Supplementary Figure S2I). In summary, due to the false fluorescence signal as a result of cell aggregation, IncuCyte data was not as conclusive as data obtained by flow cytometry, which demonstrated that the 5:1 PEI:DNA ratio was optimal for the two-plasmid intein system. This optimal ratio for the transfection mix was, therefore, used in the following experiments.

The final stage of optimization compared two alternative transfections: forward (when transfection mix is added to adherent cells seeded 24 h prior) and reverse (when transfection mix is added to the cell suspension, which is then plated and allowed to adhere). Both transfection approaches were carried out with equal amounts of plasmid DNA and PEI:DNA ratio. The level of fluorescence in cells transfected with pGFP using the forward method (Supplementary Figure S3B) was lower than that in the cells transfected with the reverse method (Supplementary Figures S3C, F). A similar result was observed for the intein-mediated GFP assembly after the trans-splicing reaction with the reverse method, resulting in 1.6 times higher fluorescent intensity (253 green objects per image) as compared to the forward (158 green objects per image) transfection (Supplementary Figure S3F). Flow cytometry analysis further confirmed data obtained in IncuCyte: 17.6% of cells transfected by the forward method were GFP+, as compared to 35.4% of GFP+ cells post-reverse transfection (Supplementary Figure S3G); therefore, the efficiency of intein trans-splicing doubled with the reverse transfection protocol. Mean fluorescent intensity values were 2.6 times higher for the reverse transfection (MFI of 39898) than those for the forward method (MFI of 15484). In summary, the reverse transfection approach increased both the transfection efficiency and intein-mediated protein assembly almost twofold. The following experiments relied on the reverse transfection with 2 µg of plasmid DNA and a 5:1 PEI:DNA ratio.

### 3.4 Comparison of intein activity

**FIGURE 5:**
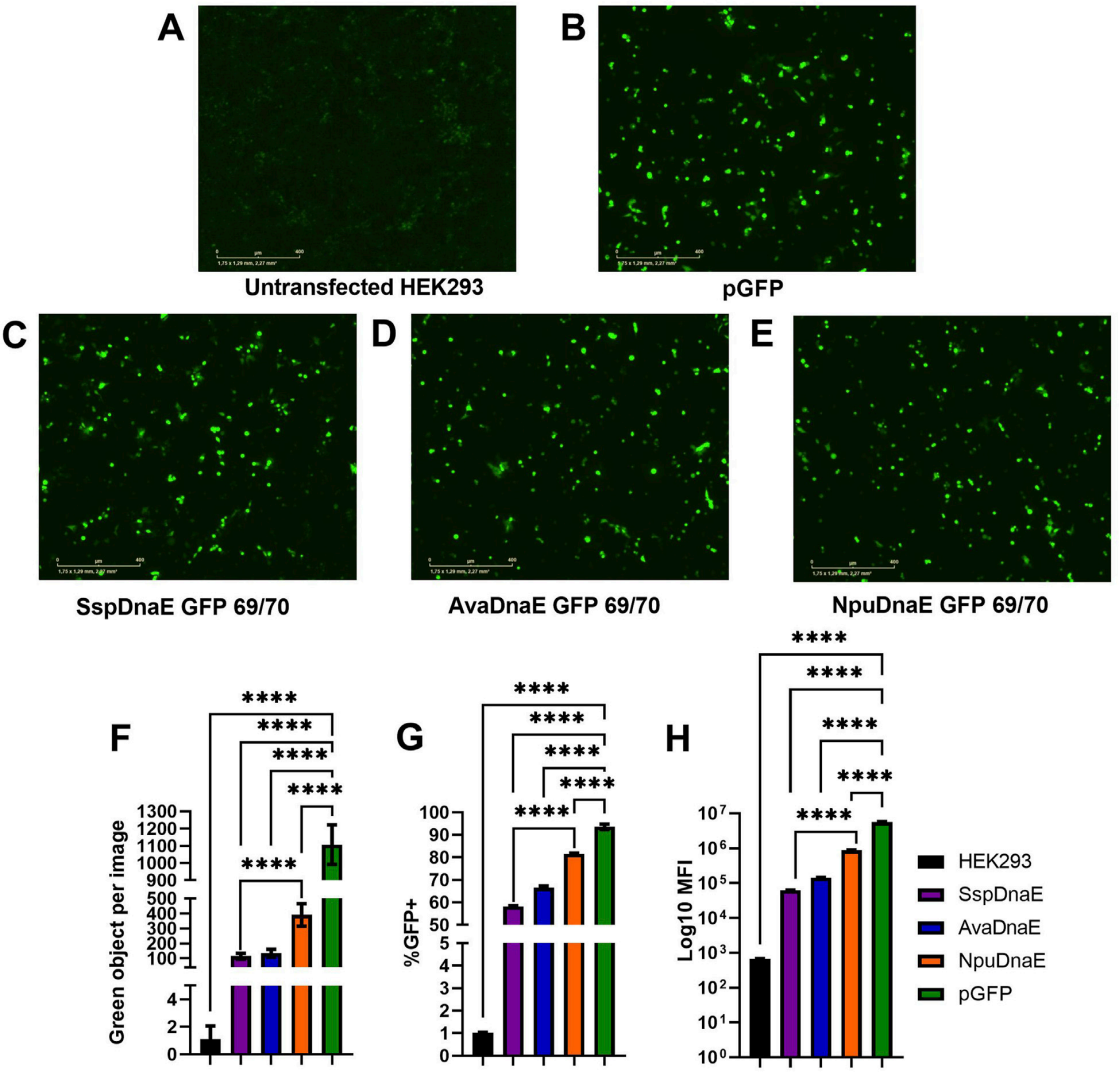
Comparison of trans-splicing activity of the Ssp, Ava, and Npu inteins. **(A–E)** Fluorescence microscopy by IncuCyte 48 h post-reverse transfection with 1 µg of each plasmid DNA at a 5:1 PEI:DNA ratio. **(F)** Comparison of fluorescence intensity (shown as green objects per image) as a result of intein-mediated GFP assembly. **(G, H)** Flow cytometry analysis demonstrating the percentage of GFP+ cells **(G)** and mean fluorescent intensity values **(H)**. (****) p-value <0.0001.

As shown previously, inteins of the DnaE group have different kinetics of interactions between the N and C parts (Figure 2C). The similar split-sites of exteins in the DnaE group provide an advantage for comparison of activities of the Ssp, Npu, and Ava inteins. This evidence is further supported by the similarity of the main catalytic sites (N+1, 70, 72, 75, and 82, and C+19, 25, 36, and 37) and the variety of the nearest surroundings of the active sites, as presented in Figure 2D. Amino acid sequences of Npu and Ava inteins, as well as the 69/70 split site of GFP, were used to design plasmids for GFP assembly using the previously optimized transfection protocol. As presented in Figures 5A–F, the efficiency of intein-mediated GFP assembly based on the level of fluorescence intensity assessed by the IncuCyte varied for the four analyzed inteins. The efficiency of Ssp and Ava DnaE intein-mediated assembly was similar (100 green objects per image) and significantly different from the untransfected control. The Npu-mediated GFP assembly was 4-fold more efficient (400 green objects per image) than Ssp and Ava; however, it was 2.5 times lower as compared to the positive control (1100 green objects per image). Flow cytometry analysis (Figure 5G) demonstrated a similar trend: 55% of cells transfected with Ssp, 65% with Ava, and 82% with Npu inteins were GFP+ as compared to 92% of cells transfected with the positive control plasmid pGFP. It is notable that the number of GFP+ cells in samples transfected with Npu intein is quite high and similar to the positive control pGFP. The mean fluorescent intensity values, shown in Figure 5H, showed a similar pattern, with Ssp-mediated GFP assembly being the least efficient (MFI of 7 × 10^4^) and Npu being highly efficient (MFI of 8 × 10^5^). The tenfold difference between Ssp and Npu indicates a significant difference in kinetic parameters that is correlated with the results from similar experiments (Shah et al., 2012). All observed differences were statistically significant (p-value <0.0001). Based on these results, the Npu intein was used for further assessment of the AAV-delivered intein-extein gene parts.

### 3.5 Western blotting of precursor and spliced molecules

**FIGURE 6:**
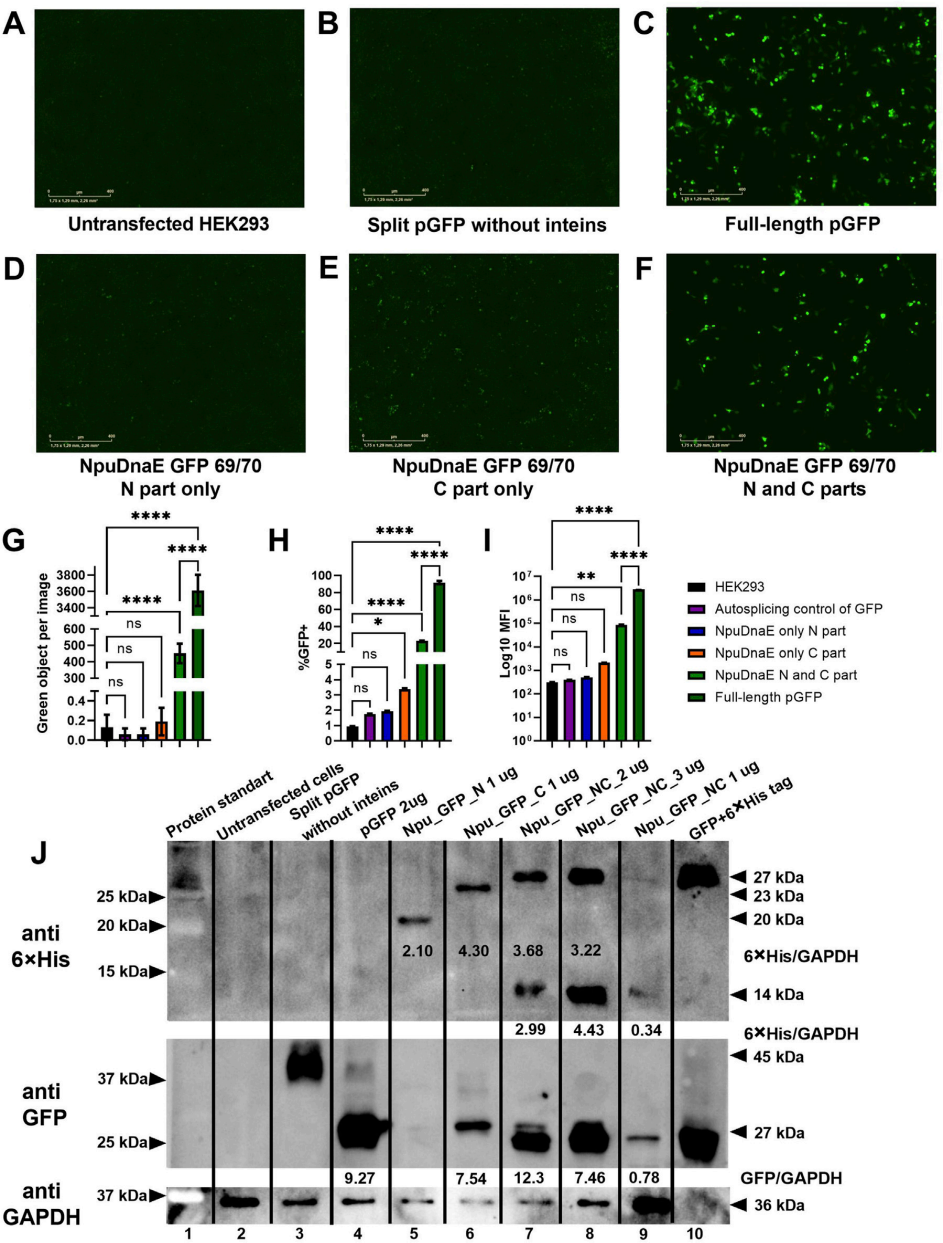
Evaluation of intein-mediated assembly. **(A–F)** Fluorescence microscopy of HEK293 cells 48 h post-transfection with combinations of plasmids as indicated. **(G)** - green object analysis of 16 images of each well with IncuCyte software, **(H, I)** flow cytometry analysis, **(J)** - Western blotting analysis with specific antibodies: upper panel - membrane probed with anti-6 × His-tag antibody, middle panel - membrane probed with anti-GFP, lower panel - membrane probed with anti-GAPDH. not significant (ns) p > 0.05, (*) p-value <0.1, (**) p-value <0.01, (****) p-value <0.0001.

Plasmids encoding NpuDnaE and GFP with a 69/70 split site were modified by the addition of a 6 × His peptide tag to the C-terminal parts. Additionally, a construct with GFP parts lacking both inteins and tags was produced to assess the possible self-assembly, described previously by Cabantous et al., in 2004. Therefore, the cells were transfected with various combinations of plasmids, as shown in Figure 6. While no GFP expression was observed in the negative control (Figure 6A) and cells transfected with either two plasmids containing parted GFP at the 69/70 site without intein attachment or single GFP parts (Figures 6B–E), co-transfection of plasmids encoding N and C GFP parts resulted in visible fluorescence (Figure 6F). Image analysis using IncuCyte software further confirmed that only the N- and C-GFP part-encoding plasmid combination resulted in intein-mediated GFP assembly with fluorescence intensity significantly different from untransfected cells and cells transfected with either a GFP part or parted GFP lacking inteins (Figure 6G). Results of flow cytometry analysis shown in Figure 6H confirmed that co-transfection of plasmids encoding the N- and C-GFP parts resulted in GFP assembly in 20% of cells, which was significantly different (p-value <0.0001) compared to the negative control (untransfected cells) and the 80% of GFP+ pGFP-transfected cells. The median fluorescence intensity of GFP assembled from N and C parts was 1 × 10^5^ (Figure 6I). To summarize, it was confirmed that assembly of GFP split at the 69/70 position is impossible without the presence of inteins.

In addition to the live imaging microscopy and flow cytometry data, Western blotting further confirmed the assembly of GFP protein of the correct molecular weight. 6 × His-tagged-polypeptides of the expected size before and after the splicing reaction were detected with anti-6 × His tag specific antibodies (upper panel). As expected, transfection of plasmids encoding N or C parts of GFP with inteins led to expression of a full-length GFP (27 kDa) and the intein (14 kDa) (lanes 7–9). Co-transfection of plasmids encoding GFP parts without inteins did not lead to GFP assembly (lane 3) and was used as a self-splicing control. The purified His-tagged GFP produced in bacteria was used as a positive GFP control (lane 10). The anti-GFP antibody (lower panel) detected the C-terminal GFP part (amino acids 70–238; lane 6) and the full-length assembled protein (lanes 7–9). The densitometry analysis was carried out with ImageJ software and used for normalization against the housekeeping GAPDH protein. The highest GFP expression was observed as a result of intein-mediated GFP assembly in cells transfected with 2 µg of total plasmid DNA (lane 7), followed by 3 µg (lane 8) and 1 µg (lane 9). It was obvious that 1 µg of total plasmid DNA was not sufficient for efficient GFP assembly. Interestingly, a misfolded product of parted GFP with incorrect conformation and antibody epitope was detected as a 45 kDa band with anti-GFP antibody (lower panel, lane 4). The nature of the used GFP antibody is such that it can detect a bigger C-terminal part of GFP but not the smaller N-terminal part. Therefore, the C and N-terminal GFP parts were additionally detected with the help of an anti-His antibody. While quantitative protein production was not the main goal of this study, Western blotting analysis clearly demonstrated a limitation of the trans-splicing reaction, which will be further investigated in future research. GFP assembly with Npu intein was 25% less effective than direct expression, but it has the highest kinetic activity and efficiency, making it suitable for the dual AAV delivery system.

### 3.6 Evaluation of AAV transduction efficiency

Recombinant AAV5 virus delivering GFP was used to optimize viral transduction of HEK 293 cells. 3 × 10^5^ cells were transduced with increasing viral doses: 5 × 10^3^, 1 × 10^4^, 2 × 10^4^, 5 × 10^4^, 1 × 10^5^, 2 × 10^5^ (six replicates).

As presented in Supplementary Figure S4, the level of green fluorescence gradually increased with an increase in viral dose (Supplementary Figures S4B–G). Image analysis with IncuCyte software (Supplementary Figure S4I) showed the increase in the number of green objects proportionate with viral doses: 22 (5 × 10^3^ Vg/cell), 28 (1 × 10^4^ Vg/cell), 48 (2 × 10^4^ Vg/cell), 147 (5 × 10^4^ Vg/cell), 214 (1 × 10^5^ Vg/cell), and 327 (2 × 10^5^ Vg/cell). Based on this data, the minimum recommended viral dose sufficient for detection of GFP assembly and fluorescence is 2 × 104 Vg/cell. Flow cytometry analysis (Supplementary Figure S4J) further confirmed these results. As can be seen in Supplementary Figure S4H, mean fluorescent intensity values increased with viral dose: 1889 (5 × 10^3^ Vg/cell), 3809 (1 × 10^4^ Vg/cell), 8,967 (2 × 10^4^ Vg/cell), 22115 (5 × 10^4^ Vg/cell), 35769 (1 × 10^5^ Vg/cell), and 51520 (2 × 10^5^ Vg/cell). The number of GFP+ cells increased gradually and could be approximated with a 4-parameter logistic dose-response curve with an R^2^ = 0.995 value: 7.2 (5 × 10^3^ Vg/cell), 13.7 (1 × 10^4^ Vg/cell), 38.7 (2 × 10^4^ Vg/cell), 58.6 (5 × 10^4^ Vg/cell), 72.4 (1 × 10^5^ Vg/cell), and 80.2 (2 × 10^5^ Vg/cell). Viral doses in the range of 3.5 × 10^4^–1 × 10^5^ Vg/cell allow to transduce HEK293 with at least 50% efficiency. Finally, a 1 × 10^5^ Vg/cell dose was used as optimal in the following experiments with inteins and AAV5 on three human cell lines to test the efficiency of trans splicing assembly in different tissues.

### 3.7 Evaluation of intein trans-splicing post-AAV5 transduction

Two human cell lines: HEK293, ARPE19 and FbP13 cells were used to assess trans-splicing efficiency following AAV5-mediated delivery of GFP parts fused to inteins. AAV5 delivering the full-length GFP (AAV5 GFP) was used as a positive control. The optimal dose of 1 × 10^5^ Vg/cell of each AAV5 was used to transduce each cell line.

**FIGURE 7:**
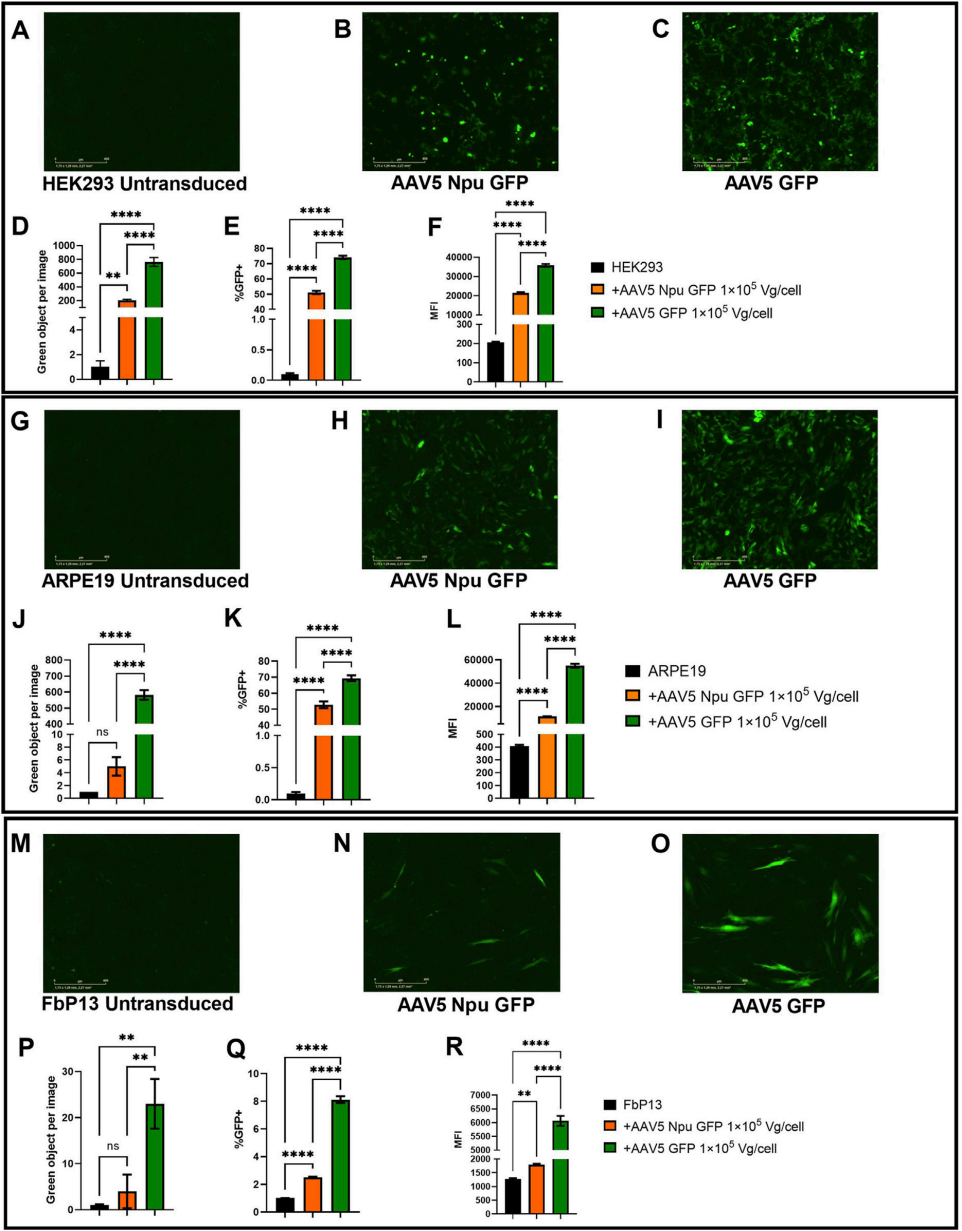
Assessment of GFP assembly post-cotransduction with AAV5 delivering protein parts. **(A–C)** HEK293 cells 72 h post-transduction. Analysis of fluorescence intensity with IncuCyte software in HEK293 **(A–D)**, ARPE19 **(G–J)**, and FbP13 **(M–P)** cells. Flow cytometry analysis of GFP+ cells and mean fluorescence intensities in HEK293 **(E, F)**, ARPE19 **(K, L)**, and FbP13 **(Q, R)** cells. not significant (ns) p-value >0.05, (**) p-value <0.01, (****) p-value <0.0001.

The levels of green fluorescence intensity were analyzed by live cell microscopy with IncuCyte and 72 h post-transduction the number of GFP+ cells and MFI values were assessed by flow cytometry. As shown in Figures 7A–C, no fluorescence was observed in the negative control, while the levels of fluorescence in the transduced cells differed by about 2.5 times (Figure 7D). A 20% difference was observed between HEK293 cells transduced with AAV5 GFP (72% GFP+ cells) and AAV Npu GFP (52%) delivering GFP parts fused to inteins (Figure 7E). Mean fluorescence intensity values were 1.6-fold higher in AAV GFP-transduced cells as compared to intein-mediated GFP assembly. The differences observed were statistically significant (p-value <0.0001).

ARPE19 cells were used as a more relevant cell model for ophthalmologic genetic disorders. Following AAV transduction, the level of fluorescence intensity in cells with intein-assembled GFP was 150 times lower than that in cells transduced with AAV5 GFP (Figure 7J). Despite this, 49% of AAV Npu GFP and 71% of AAV5 GFP-transduced ARPE-19 cells were GFP+ as shown by flow cytometry analysis (Figure 7K). As expected, MFI values in AAV GFP-transduced cells were 3 times higher than those in cells transduced with AAV Npu GFP. The observed differences were statistically significant (p-value< 0.0001). AAV5 Npu GFP transduction of primary patient fibroblasts FbP13, demonstrated that these cells are not only significantly less susceptible to AAV transduction, but also, as a consequence, to efficient co-transduction needed for GFP assembly. IncuCyte analysis shown in Figure 7P, showed no statistically significant difference between the untransduced negative control and cells with assembled GFP, where green fluorescence intensity was 4 times lower than that observed in cells transduced with AAV delivering the full-length GFP. 2.5% of AAV5 Npu GFP-transduced and 6% of AAV5 GFP transduced FbP13 cells were GFP+ positive, as shown by flow cytometry analysis (Figure 7Q). MFI values presented in Figure 7R showed a 3-fold difference between assembled and the full-length GFP and a 2-fold difference between assembled GFP and the negative control. Despite the low level of fluorescence intensity compared to HEK293 (Figures 7A–C) and ARPE-19 cells, (Figures 7G–I) GFP expression was nevertheless observed in FbP13 cells (Figures 7M–O) post AAV5 transduction.

## 4 Discussion

Achieving successful large protein trans-splicing is essential for overcoming AAV packaging limitations, as described in numerous articles previously (Tornabene et al., 2019; Esposito et al., 2022; Li et al., 2023; Kolesnik et al., 2024; Tasfaout et al., 2024). Literature analysis revealed that the majority of unique AAV-based therapies relying on split intein technology have been used for the treatment of hereditary retinopathies. Clinical manifestations of these disorders appear in approximately two dozen major phenotypic variants of blindness caused by mutations in 300 different genes (Retnet, 2024). A number of IRD genes exceed AAV packaging capacity; these include genes such as *ABCA4, CEP290, EYS, MYO7A,* and *USH2A*, with the most frequently appearing mutations (Pulman et al., 2022). The intein technology has already been successfully applied on more than one occasion for *ABCA4, CEP290*, and *MYO7A* genes (Tornabene et al., 2019; Li et al., 2023), which are not exceeding 8 kilobases in length. However, no solution has been found so far for *EYS* (10.8 kilobase) and *USH2A* (18.9 kilobase) due to theirs giant sizes. The intensified focus on ophthalmologic disorders can be explained by a number of factors, which include the relatively compact size of the organ, isolation from the general immune system, and the precise localization of the affected tissues (Minskaia et al., 2023). In contrast, muscle, which is the target for Duchenne dystrophy therapy, is characterized by the opposite set of properties. The number of studies using separate delivery of dystrophin parts is increasing, with each new iteration improving the results (Li et al., 2008; Tasfaout et al., 2024). There are also new developments in therapies targeting hemophilia A (Esposito et al., 2022). Furthermore, the application of intein-mediated delivery technology has now been extended to genome editors. The combination of genome editors with inteins will significantly extend the list of genetic diseases that can be effectively treated. Furthermore, the ongoing advancement in the direct delivery of divided genes using interines will potentially facilitate the registration of a drug that employs this technology.

Optimization of transfection and transduction protocols can significantly increase trans-splicing efficiency in a two-plasmid system, save reagents, and improve experimental outcomes. Transfection can be optimized in several ways: the total amount of DNA, the molar ratio of transfection reagent to DNA, the time of complex formation, and the interface of the transfection complex with cells (transfection of cell suspension vs. adherent cells) (Supplementary Figures S1–S3). The most significant improvement was achieved by using a reverse transfection protocol (transfecting cells in suspension and then plating to allow for adherence) with an increased number of cells. The efficiency of the trans-splicing reaction increases proportionally to the level of precursor expression. In this study, 1 μg of each plasmid for the two-plasmid approach was optimal for trans-splicing assembly. From Western blotting analysis, we have seen that usage of equimolar and equal mass amounts of pDNA leads to unequal expression of N and C proteins. As the C part also plays a supporting role in reaction, it stops when all N parts react. Thus, we recommend using the mass ratio of N to C in proportion 2:1 to compensate for the difference in expression level and reduce the amount of unspliced products. However, this parameter may change as the number of plasmids increases and must be optimized experimentally in each specific case. Following optimization of the transfection protocol, intein-mediated GFP assembly was significantly improved and resulted in 82% of GFP+ transfected HEK293 cells as compared to 92% of cells transfected with the positive control pGFP (Figure 5).

The site of protein split is also crucial for effective protein assembly. The amino acid composition of the splice site is unique for each group of inteins, as it contains the primary catalytic site at the C + 1 position that initiates the reaction, and the reaction enhancers are located six amino acids around it (Cheriyan et al., 2013). In addition, protein secondary structures affect the recovery of activity and can render the protein non-functional with the restored structure, as demonstrated for the 47/48 split site (Figure 4). Large protein complexes do not have a crystallized structure, and therefore, the influence of secondary structure can only be assessed experimentally Thus, each predicted target protein partition site must be verified using both the activity test and Western blotting. We recommend testing at least three cleavage sites with the predicted highest score after sequence analysis and a fixed cysteine at position C + 1 for DnaE group inteins, as was done in this study and by Li and colleagues (Li et al., 2023). The most effective site can be applied to inteins in one structural group, as was shown in Figure 4. A final recommendation for plasmid design is to tag the C-terminus of each protein precursor with immunogenic peptides such as 6 × His, Myc, and HA. Detection of protein parts with anti-tag antibodies as well as the full-length target protein with specific antibodies by Western blotting is highly recommended (Tornabene et al., 2019).

The difference in splicing mediated by the SspDnaE and NpuDnaE inteins (Figure 5) is explained by the addition of 20 amino acids at the C-terminus of N part. Excision of the N-part plays a primary role in the trans-splicing reaction, leading to a faster interaction due to the lower molecular weight. In contrast, the differences between Ava DnaE and Npu DnaE cannot be easily explained. The similar size of the N- and C-parts indicates that kinetic properties are determined not only by the size but also by more complex factors such as amino acid composition, their total charge and final conformation. Sequence alignment of ten DnaE inteins showed 100% similarity in the active domains and catalytic sites and a minute difference of several amino acids in the immediate environment. The most frequent substitutions are observed at positions N+25, 27, 35, 38, 52, 58, 64, and 76 in the N-part and C + 6, 14, 21, 22, 27, and 32 in the C-part, which can be used to further analyze the effect of mutations on the reaction kinetics. In addition, most of these positions were previously used (Stevens et al., 2016) to design Cfa-intein, which increased trans-splicing activity 2-fold. However, methods for improving intein trans-splicing are not limited to evolutionary mutagenesis, as confirmed by Xia H.F. and colleagues via molecular docking (Xia et al., 2022). Other computer modeling techniques, such as modeling the interaction dynamics of protein parts, can provide a lot of valuable information for future research.

In addition to the successful GFP assembly post-plasmid transfection, co-transduction of HEK293 and ARPE19 cell lines with AAV5 delivering GFP parts at 1 × 10^5^ Vg/cell led to 51% and 53%, respectively, of GFP+ cells as a result of intein-mediated trans-splicing assembly as compared to 74% of GFP+ cells transduced with the positive control AAV5 delivering the full-length GFP (Figure 7). In other studies, the same inteins were tested in HEK293 cells post-AAV8 transduction (Tornabene et al., 2019; Esposito et al., 2022; Li et al., 2023), in ovarian cancer HeLa cells post-AAV8 transduction (Tornabene et al., 2019), in fibrosarcoma HT1080 cells post-AAV2 transduction (Ferreira et al., 2023), and in hepatocellular carcinoma HepG2 cells post-AAV8 transduction (Padula et al., 2022). Therefore, the successful intein-mediated assembly of the protein delivered by AAV5 co-transduction of HEK293 and ARPE19 cells is presented for the first time. The high level of transduction efficiency and splicing assembly in ARPE19 cells could be further used for developing cellular models of hereditary retinal diseases. However, as shown in Figure 7, the transduction efficiency of FbP13 primary fibroblasts with AAV5 was significantly lower: only 8% and 2.5% of cells were GFP+ after AAV-GFP or co-transduction and splicing of split GFP, respectively. Transduction with AAV serotype 6, however, could solve this problem if necessary, as previously reported (Larin et al., 2024).

Efficiency of viral transduction can be improved by optimizing viral doses or by specific capsid modification, which improves the interaction with target cell receptors, as was demonstrated previously (Khabou et al., 2016; Chatterjee et al., 2022; Gurtsieva et al., 2024). It is worth remembering that each virus preparation has its own dose response curve, which depends on the ratio of empty to full capsids, time, and storage conditions. Six increasing doses were used to determine the optimal amount of virus (Supplementary Figure S4). Cytometry analysis showed that the saturation point was achieved at 1 × 10^5^ Vg/cell, resulting in 78% of GFP+ cells. Similar results were demonstrated by Ferreira and colleagues (2022) using AAV2 with the NpuDnaE intein on the HT1080 cancer line. In this study, the low dose of 4 × 10^4^ Vg/cell was only effective for transduction of fibrosarcoma cells, resulting in 95% of GFP+ cells; however, it is worth mentioning that cancer cells are more susceptible to viruses, which lowers the effective dose sometimes by several orders of magnitude. Similar results were obtained in this study in non-cancerous HEK293 cells with AAV5 and NpuDnaE inteins.

The data presented in this study can be used as a basis for further studies for various applications. The described method relying on DnaE inteins can be applied to many other proteins, such as therapeutic (Li et al., 2023; Esposito et al., 2022; Tornabene et al., 2019), primer complex (Davis et al., 2024), Cre recombinase (Wang et al., 2012), and many others. AAV5 itself as a gene delivery tool can be used in the treatment of a wide range of hereditary diseases due to its low immunogenicity (Klamroth et al., 2022) and wide tissue tropism (Srivastava, 2016).

## Data Availability

The original contributions presented in the study are included in the article/Supplementary Material, further inquiries can be directed to the corresponding author.
