# Supplementary material for: Protein trans-splicing: optimization of intein-mediated GFP assembly as a model for the development of gene therapy
Source: Front Bioeng Biotechnol. 2024 Nov 20;12:1488912. doi: 10.3389/fbioe.2024.1488912 (PMC11614648; doi:10.3389/fbioe.2024.1488912)
Supplement: Supplementary file 3 [file DataSheet1.PDF]

# *Supplementary Material*

## Supplementary Figures

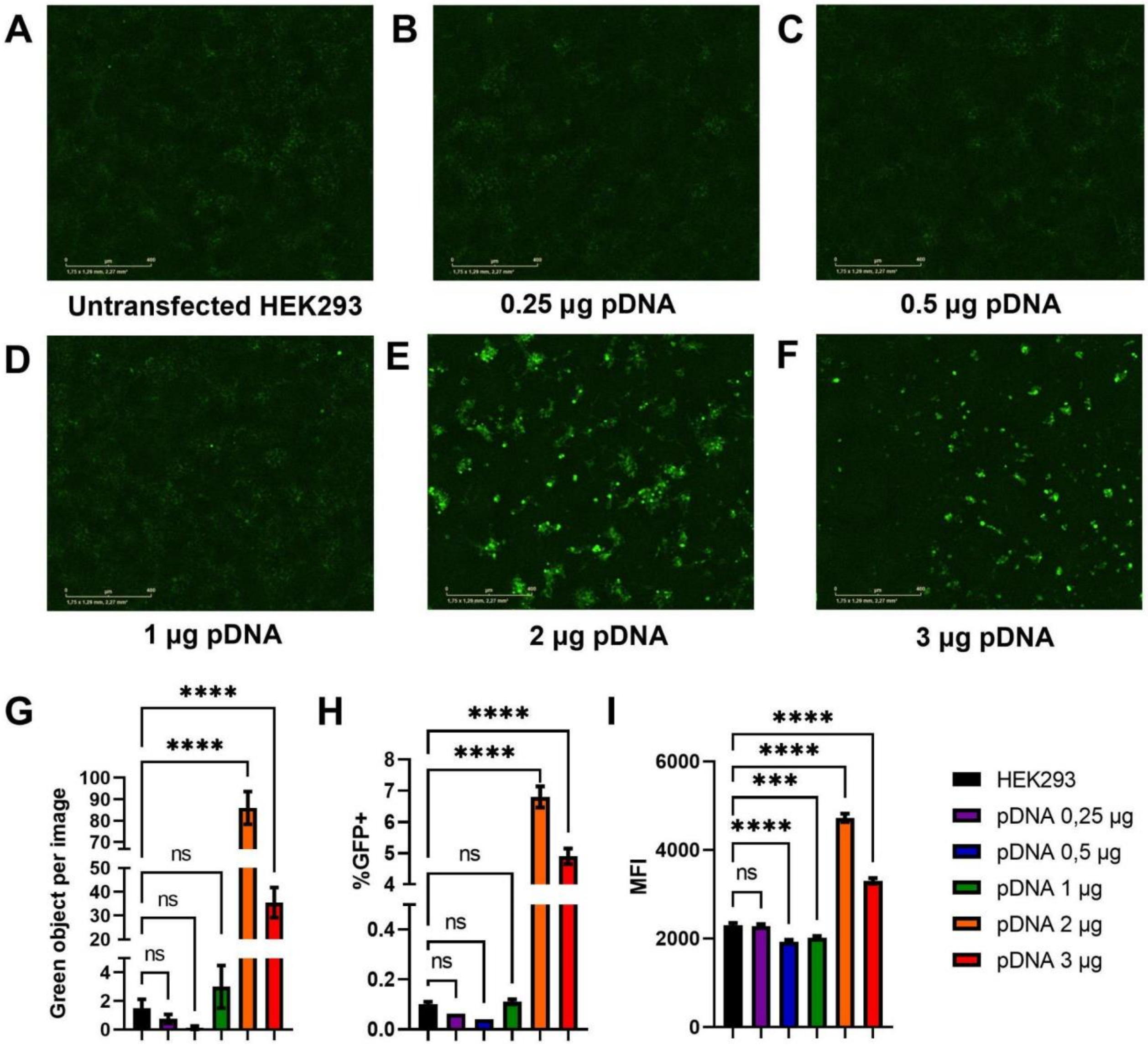


**Supplementary Figure 1.** Effect of various amounts of transfected plasmid DNA (SspDnaE-GFP-69/70) on GFP trans-splicing reaction. A-F. IncuCyte fluorescence microscopy of HEK293 cells at 48 hours post-transfection with various amounts of total DNA and fixed PEI:DNA ratio of 2:1. G. IncuCyte analysis of images. H and I. Flow cytometry analysis at 72 hours post-transfection. Not significant (ns) p-value > 0.05, (*) p-value < 0.05, (****) p-value < 0.0001.

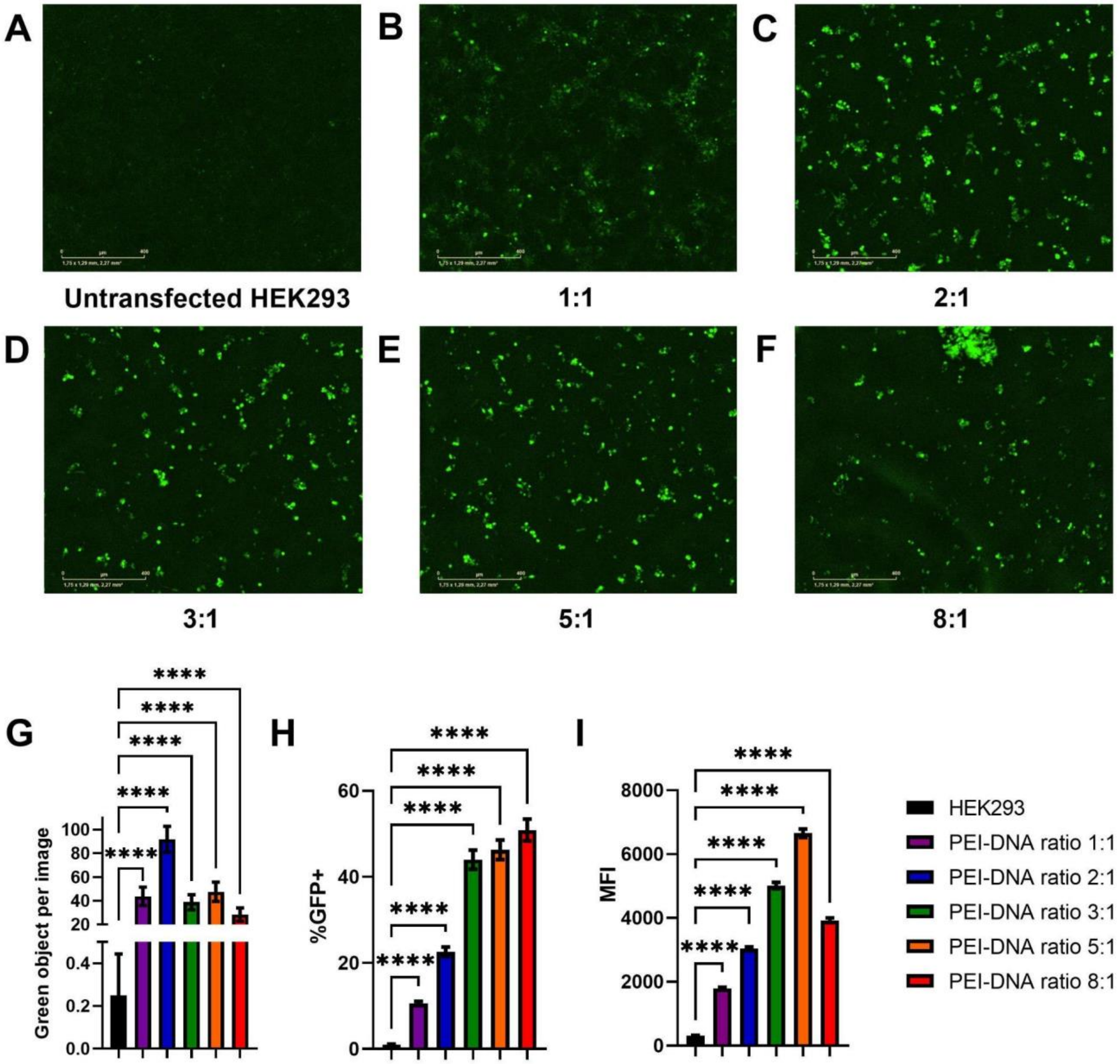


**Supplementary Figure 2.** Effect of PEI-DNA ratio on SspDnaE-GFP-69/70 trans-splicing reaction. A-F. GFP fluorescence intensity was detected by IncuCyte microscopy of HEK293 cells 48 hours post-transfection with 2 μg of total DNA and various PEI:DNA ratios. G. IncuCyte analysis of images: the level of fluorescence intensity is defined by green objects per image. The number of GFP+ cells (%GFP, H) and their mean fluorescence intensity values (I) were analyzed by flow cytometry 72 hours post-transfection. Not significant (ns) $p > 0.05$, (****) p-value $< 0.0001$.

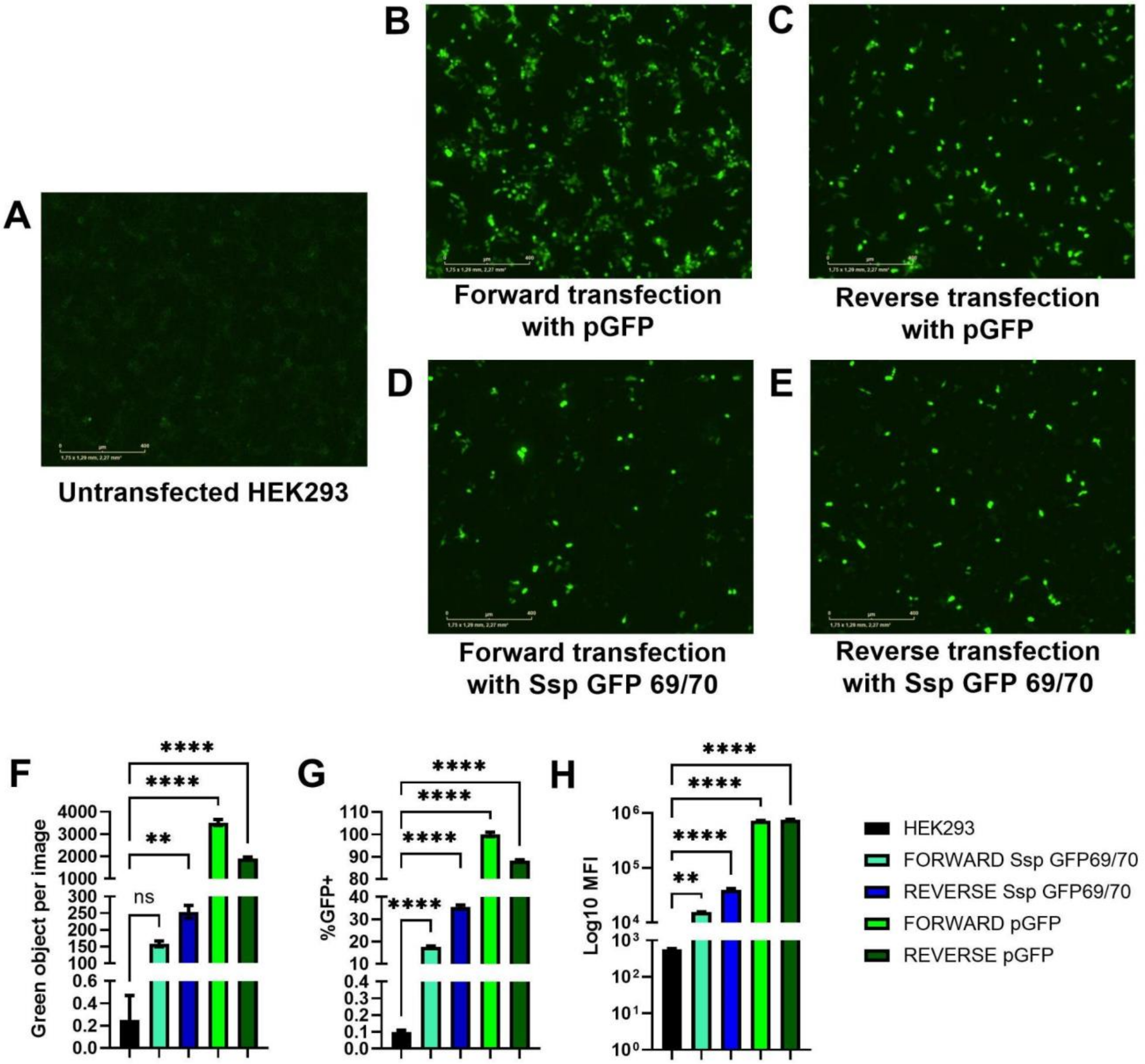


**Supplementary Figure 3.** Effect of transfection procedure on protein trans-splicing. A-F. Fluorescence microscopy by IncuCyte of HEK293 cells 48 hours post-reverse and forward transfection with 2 μg of SspDnaE-GFP-69/70 (5:1 PEI:DNA ratio). F. IncuCyte analysis of images. The level of fluorescence intensity is measured in green objects per image. G and H. Flow cytometry analysis demonstrating the percentage of GFP+ cells (G) and mean fluorescence intensity (H). not significant (ns) $p > 0.05$, (**) p-value < 0.01, (***) p-value < 0.001, (****) p-value < 0.0001.

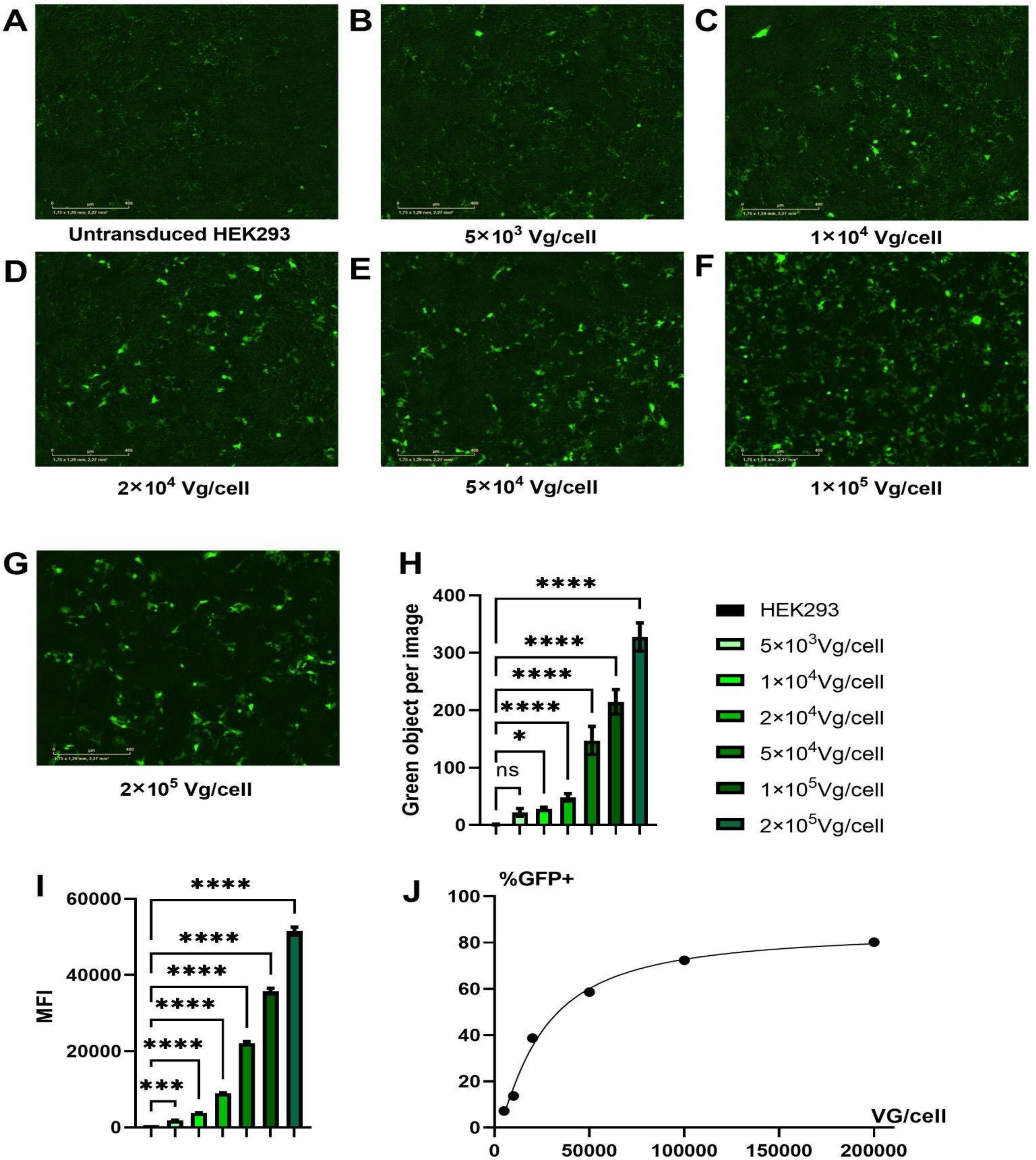


**Supplementary Figure 4.** Evaluation of AAV5 GFP transduction efficiency. A-F. Fluorescence microscopy of HEK293 cells 72 hours post-transduction with increasing doses of AAV5 GFP. H - IncuCyte analysis of green fluorescence intensity (green objects per image). I. Flow cytometry analysis: MFI values of each sample and the dose response curve for GFP+ cells (J). not significant (ns) p-value > 0.05 (*) p-value < 0.05, (**) p-value < 0.01, (***) p-value < 0.001, (****) p-value < 0.0001.

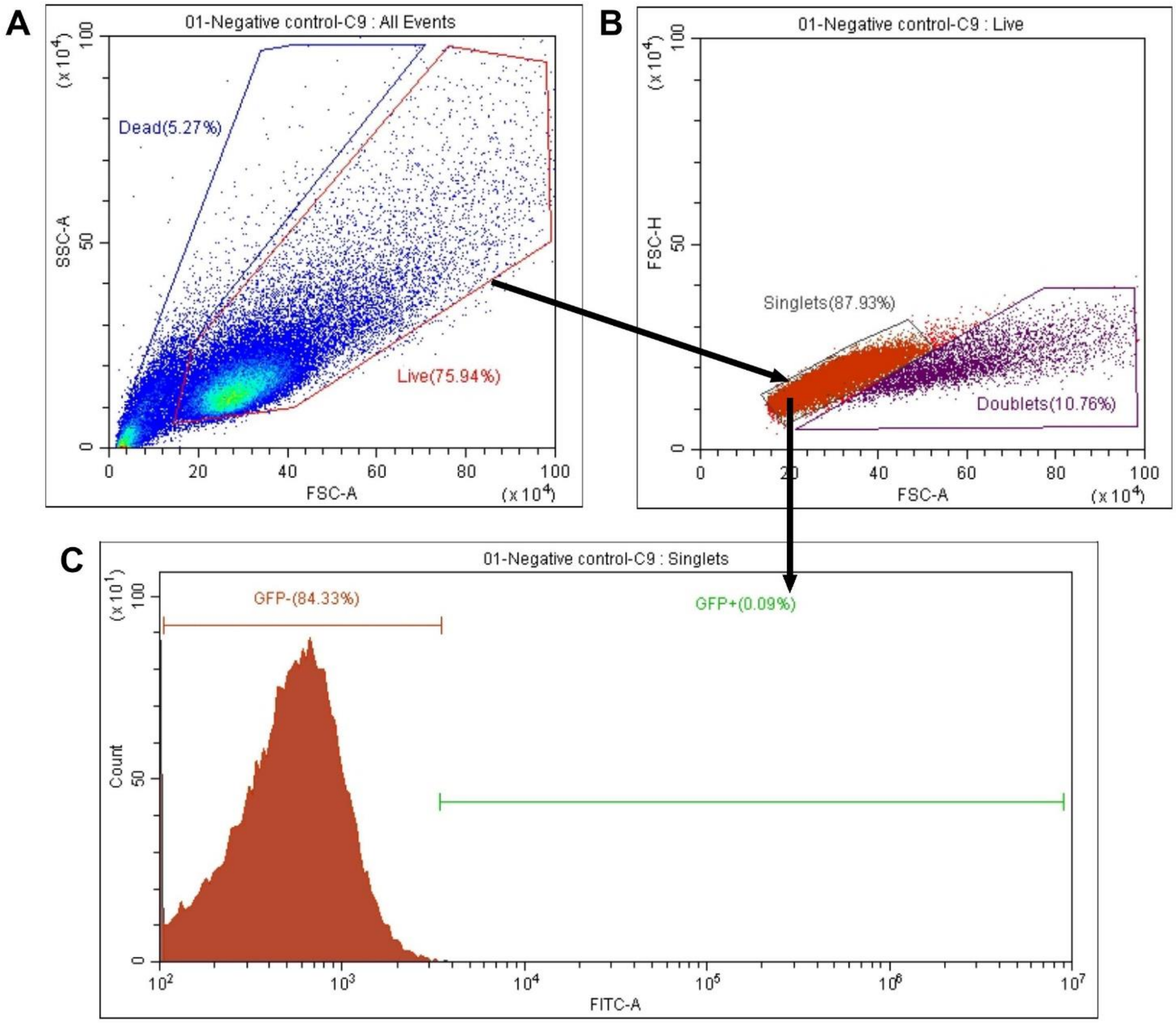


**Supplementary Figure 5.** Gating strategy of flow cytometry analysis (untransfected HEK293 cells): A – Live/dead analysis of all cells; B – live singlets/doublets analysis; C – live singlets GFP negative population analysis.

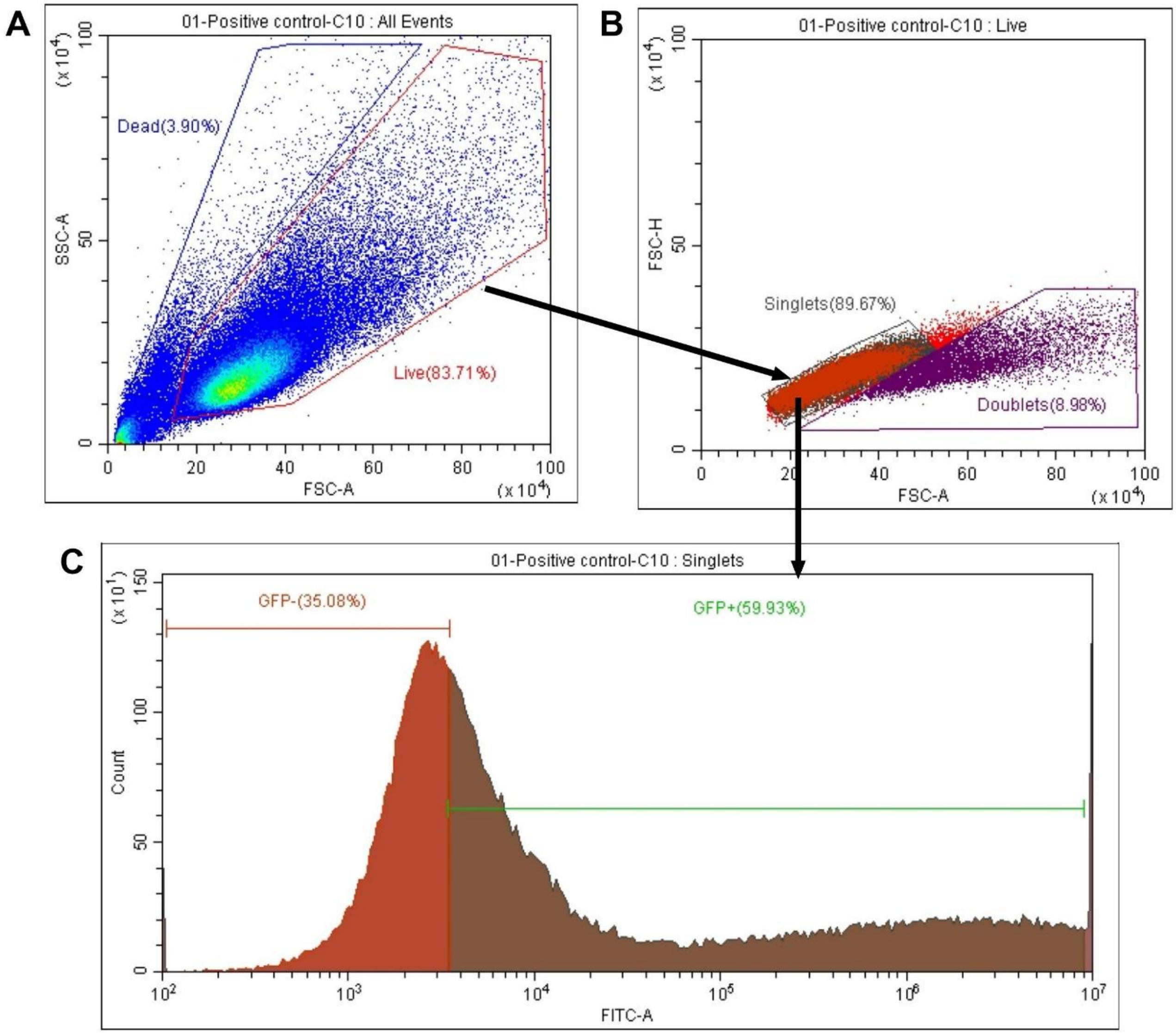


**Supplementary Figure 6.** Gating strategy of flow cytometry analysis (HEK293 cells transfected with pAAV-CMV-GFP plasmid): A – Live/dead analysis of all cells; B – live singlets/doublets analysis; C – live singlets GFP positive population analysis.
